# A Novel Prediction-Optimization Machine Learning Framework for Nanofluid-Based Photovoltaic/Thermal Systems

**DOI:** 10.3390/nano16110680

**Published:** 2026-05-30

**Authors:** Chengyuan Li, Yankai Huang, Zheng Zhang, Yan Zhou, Ruipeng Geng, Chengchao Wang, Lanxin Ma

**Affiliations:** 1 School of Nuclear Science, Energy and Power Engineering, Shandong University, Jinan 250061, China; 2 The Chinese People’s Liberation Army Troop 32214, Nanjing 211113, China; 3 School of Energy and Power Engineering, Nanjing University of Aeronautics and Astronautics, Nanjing 210016, China

**Keywords:** nanofluids, photovoltaic/thermal systems, machine learning, deep neural network, SHAP analysis, genetic algorithm optimization

## Abstract

Nanofluid-based spectral filtering offers a promising approach to enhance photovoltaic/thermal (PV/T) system performance by utilizing the full solar spectrum. However, system optimization remains challenging due to complex nonlinear relationships between nanofluid parameters and overall performance. This study develops a prediction-optimization framework integrating deep neural networks (DNN) with genetic algorithms (GA) to accurately analyze multi-parameter interactions and achieve globally optimal designs for nanofluid-based PV/T systems. High-throughput datasets for three nanofluids (Ag, Au, Al) were constructed using theoretical calculations that combined Lorentz–Mie theory, Monte Carlo simulations, and a coupled opto-electro-thermal model. Three machine learning models—DNN, random forest (RF), and decision tree (DT)—were employed to predict key PV/T performance parameters. By synergizing machine learning with GA, a closed-loop prediction-optimization process was established to efficiently identify optimal design parameters. Among the models evaluated, the DNN demonstrated superior performance, achieving prediction accuracies above 99.48% for all three key performance indicators (*η*_pv_, *η*_th_, and MF), significantly outperforming the RF and DT models. Furthermore, SHAP analysis was conducted to quantify the contribution of each input feature and enhance model interpretability. Coupled with the GA, the DNN-GA framework successfully identified globally optimal design parameters for each nanofluid. For instance, for Ag nanofluid, the optimal combination (*r* = 4.02 nm, *h* = 9.91 mm, *f*_v_ = 9.45 × 10^−5^) yielded a maximum MF value of 1.3603. This work presents an innovative machine learning framework for designing nanofluid filters in PV/T systems, which reduces reliance on iterative experimentation and accelerates the development of high-performance solar energy systems, demonstrating practical value.

## 1. Introduction

**Figure 1:**
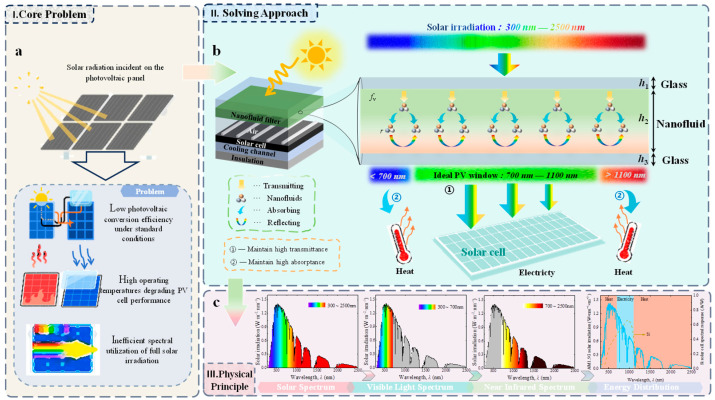
(**a**) The core problem of the solar photovoltaic system. (**b**) Schematic diagram of the PV/T System. (**c**) AM1.5D solar irradiation spectrum, the spectral response of a silicon solar cell, and the distribution of solar spectral energy for separate conversion into electricity and heat.

Within the backdrop of the global energy crisis, the development of renewable energy has accelerated due to its critical role in mitigating greenhouse gas emissions and enhancing energy security [1]. Among various renewable sources, solar energy stands out for its inexhaustible and sustainable nature [2], driving the transition of energy systems from high-carbon to low-carbon alternatives. Photovoltaic (PV) technology [3,4], which directly converts solar radiation [5] into electricity, represents one of the most widely deployed solar energy utilization pathways. However, as shown in Figure 1a, conventional PV systems suffer from low photovoltaic conversion efficiency [6], being only capable of efficiently converting photons within a specific spectral range into electrical energy [7]. Radiation outside this range is largely converted into heat, leading to elevated operating temperatures that degrade PV cell performance [8,9]. This performance degradation constitutes a critical challenge that researchers urgently need to address.

To overcome this limitation, photovoltaic/thermal (PV/T) systems [10,11] have emerged by integrating PV modules with solar thermal collectors into a unified configuration [12]. This hybrid system enables the simultaneous generation of electricity and useful thermal energy, thereby significantly enhancing the overall utilization efficiency of solar energy [13,14]. Particularly, PV/T systems employing nanofluids as spectral beam splitters represent a highly promising technological pathway. The radiative properties of nanofluids can be precisely customized by adjusting the size, morphology, and material composition of the suspended nanoparticles [15]. As illustrated in Figure 1b, they selectively absorb longer-wavelength bands in the solar spectrum that cannot be efficiently utilized by PV cells and convert this energy into heat, while maintaining high transmittance for the shorter-wavelength bands to which PV cells are responsive—allowing this portion of sunlight to reach silicon solar cells unimpeded for photovoltaic conversion [16]. Guided by the distribution characteristics of the solar irradiance spectrum, the spectral response of silicon solar cells, and the allocated solar spectral energy for electrical and thermal conversion, as shown in Figure 1b, enables efficient full-spectrum solar energy utilization [17], effective thermal management of PV cells, and ultimately achieves comprehensive performance enhancement of PV/T systems [18].

The performance of PV/T systems is assessed by their electrical and thermal energy conversion efficiencies. The electrical efficiency is governed by the solar cell’s short-circuit current density, open-circuit voltage, and fill factor [19], whereas the thermal efficiency is determined by the heat absorption and transfer capabilities of the nanofluid. A central challenge in PV/T system optimization lies in balancing these two efficiencies to maximize overall performance, which is a task involving trade-offs. Consequently, researchers have long utilized both experimental and numerical approaches to progressively improve the performance of nanofluid-based spectral-filtering PV/T systems for enhanced solar energy utilization. In experimental studies, Otanicar et al. [20] developed a hybrid CPV/T system utilizing gold and indium tin oxide (ITO) nanoparticles, which was experimentally evaluated under ambient temperatures exceeding 100 °C. Han et al. [21] proposed a PV/T system incorporating an Ag/CoSO_4_–propylene glycol (PG) nanofluid-based optical filter, with its performance characterized under indoor laboratory conditions. Hashemian et al. [22] evaluated an Ag–Cr_2_O_3_/glycerol optical filter in terms of total exergy efficiency, merit function, and energy conversion efficiency. Through experimental testing, researchers can accurately obtain the photothermal characteristic parameters of nanofluids, providing reliable data support for the further optimization of PV/T systems. However, when key parameters of nanofluids, such as particle size, effective concentration, and nanofluid layer thickness change, experimental verification must be carried out for each parameter combination one by one, resulting in rather low research efficiency. In numerical simulation studies, Crisostomo et al. [23] developed an optical and thermal loss model to estimate the electrical and thermal output of concentrating photovoltaic/thermal (CPV/T) collectors. Ju et al. [24] developed a novel 2D-3D coupled numerical model to evaluate the optical, thermal, and electrical performance of a concentrated PV/T system integrated with a nanofluid-based spectral filter. Although numerical simulation methods reduce the reliance on physical prototypes and complex experimental setups, they often involve simplifications and assumptions that may limit their ability to fully capture the intricate multi-physics and multi-parameter interactions present in actual systems. These limitations underscore the necessity for more efficient and comprehensive approaches to navigate the complex multi-parameter design space of nanofluid-based PV/T systems.

To address the limitations of experimental validation and numerical simulation methods, numerous researchers have explored nanofluid parameters that enhance the overall performance of systems through optimization algorithms. Han et al. [25] proposed an optimization approach based on Mie scattering theory and the Lambert-Beer law, which enables the selection of the optimal particle concentration of nanofluids. Ju et al. [26] considered two categories of factors, namely nanofluid parameters and system operating parameters, and employed the Genetic Algorithm (GA) to optimize the nanofluid-based spectral splitting photovoltaic/thermal (NSS-PV/T) system. Huang et al. [27] put forward an optimization method based on the merit function (MF) to optimize Au@SiO_2_-based nanofluid filters. Despite the extensive research conducted on nanofluid optical filters in existing studies, these works generally remain at the level of parametric studies and fail to systematically investigate the full-range combinations of nanomaterial and fluid parameters. Consequently, the optimality of their proposed parameters often cannot be fully guaranteed.

The advancement of machine learning (ML) has introduced a data-driven paradigm [28] that shows significant potential for application in PV/T systems [29]. Hao et al. [30] developed a multi-performance prediction model for a PV/T-glass drying chamber coupled system using several ML architectures, including convolutional neural networks (CNNs), long short-term memory (LSTM) networks, and gated recurrent units (GRUs). Although the direct application of ML in the PV/T domain remains relatively limited to date, these methods have been extensively validated in foundational disciplines closely related to PV/T systems, such as optics, heat transfer, and materials science. For instance, Artur et al. [31] created an artificial neural network (ANN)-based approach for detecting and analyzing single optical nano-objects and their mixtures. Additionally, Xiao et al. [32] proposed an unconventional ML-based framework that offers a new pathway for the efficient development of thermal nanophotonic devices. These developments demonstrate the potential for transferring ML methodologies to PV/T systems, which is not only technically feasible but also capable of addressing core challenges in the field, such as insufficient accuracy in performance prediction and low efficiency in parameter optimization. Consequently, ML provides a novel and efficient technical pathway for the optimal design of PV/T system s.

## 2. Model and Methods

### 2.1. Overview of Machine Learning Assisted Design of Nanofluid-Based PV/T Systems

**Figure 2:**
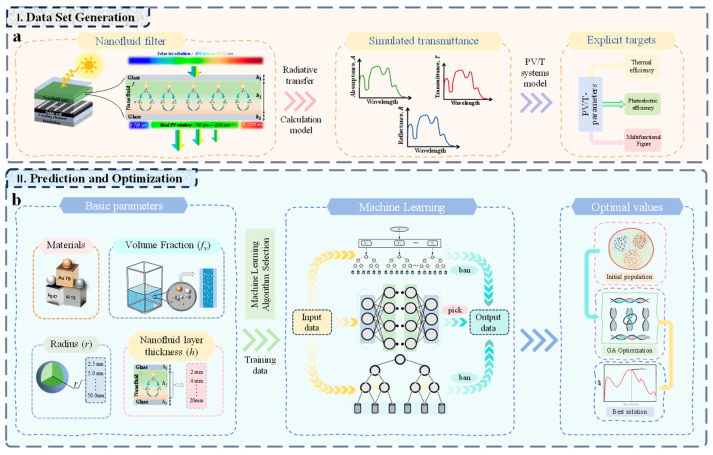
(**a**) Constructing a nanofluid dataset for machine learning training through high-throughput theoretical calculations. (**b**) Using machine learning to predict the performance of nanofluid PV/T systems, the generated prediction results are used for subsequent genetic algorithm optimization.

The optical and radiative properties of nanofluids are central to the performance of nanofluid-based PV/T systems. To systematically investigate and optimize this performance, this study develops a closed-loop “prediction–optimization” framework that integrates machine learning with a genetic algorithm, the overall workflow of which is illustrated in Figure 2. The research begins with precise physical modeling of the nanofluid-based spectral-splitting PV/T system. As shown in Figure 2a, the system consists of a water-based nanofluid layer, a glass cover, a silicon solar cell, and a water-cooling channel. To decode the complex structure–performance relationships inherent in this system, we first constructed a large-scale data set containing Ag, Au and Al nanofluids through high-throughput theoretical calculations. Specifically, by combining Lorentz–Mie theory with multi-layer Monte Carlo radiative transfer simulations, we accurately computed the spectral transmittance corresponding to various combinations of nanoparticle radius (*r*), volume fraction (*f_v_*), and nanofluid layer thickness (*h*). These optical results were subsequently fed into a coupled opto-electro-thermal mathematical model to calculate the corresponding (*η*_pv_, *η*_th_ and MF), thereby forming a comprehensive structure–performance database for machine learning training.

Subsequently, as depicted in Figure 2b, using the generated dataset, we trained three machine learning models—DNN, RF, and DT to establish an accurate mapping from the input geometric parameters to the system’s output performance metrics. Through rigorous performance evaluation and comparison, the DNN model was selected as the optimal predictor due to its superior accuracy and generalization capability, providing a core tool for rapid and reliable performance prediction.

Finally, the trained DNN model was synergistically integrated with a GA for optimization. Using the DNN as a fitness evaluation function, the GA efficiently explores the high-dimensional parameter space to rapidly identify the optimal combination of nanofluid geometric parameters that maximizes the system’s overall MF. This closed-loop prediction–optimization strategy significantly reduces reliance on traditional trial-and-error experimentation or computationally expensive numerical simulations, offering a powerful and advanced methodology for advancing the design and performance of nanofluid-enhanced PV/T systems.

### 2.2. Establishment of the Coupled Opto-Electro-Thermal Model

The radiative properties of a system of spherical nanoparticles are derived from the optical characteristics of a single nanoparticle, as defined by the Lorentz-Mie theory [33,34]. For a single spherical particle, the theory yields the extinction cross section *C*_ext_, the scattering cross section *C*_sca_, and the scattering phase function Φ_p_(*θ*) [33]:

(1) Cext=2πk12∑n=1∞2n+1Rean+bn

(2) $$C_{\mathrm{sca}}=\frac{2\pi }{{k_{1}}^{2}}\sum_{n=1}^{\infty }\left(2n+1\right)\left({\left|a_{n}\right|}^{2}+{\left|b_{n}\right|}^{2}\right)$$

(3) $$\Phi_{p}\left(\theta \right)=\frac{2\pi }{C_{\mathrm{sca}}}\left[{\left|S_{11}\left(\theta \right)\right|}^{2}+{\left|S_{22}\left(\theta \right)\right|}^{2}\right]$$

where *k*_1_ = 2π*n*_m_/*λ* defines the wave number in aqueous medium, *n*_m_ is water’s refractive index (real part), and *a*_n_, *b*_n_ represent Mie coefficients. The efficiencies follow as *Q*_sca_ = *C*_sca_/*A* (scattering) and *Q*_ext_ = *C*_ext_/*A* (extinction) for particle cross-section *A*, with *S*_11_, *S*_22_ being amplitude scattering matrix components.

For spherical nanoparticles, the Mie scattering coefficient is [33]:

(4) $$a_{n}=\frac{m\psi_{n}(mx){\psi^{\prime }}_{n}(x)-\psi_{n}(x){\psi^{\prime }}_{n}(mx)}{m\psi_{n}(mx){\xi^{\prime }}_{n}(x)-\xi_{n}(x){\psi^{\prime }}_{n}(mx)}$$

(5) $$b_{n}=\frac{\psi_{n}(mx){\psi^{\prime }}_{n}(x)-m\psi_{n}(x){\psi^{\prime }}_{n}(mx)}{\psi_{n}(mx){\xi^{\prime }}_{n}(x)-m\xi_{n}(x){\psi^{\prime }}_{n}(mx)}$$

where the particle size parameter with radius *r* is *x* = *k*_1_*r*, and *m* = *m*_p_/*m*_host_ is the relative refractive index, *m*_p_ = *n*_p_ +*iκ*_p_ and *m*_host_= *n*_m_ +*iκ*_m_ are the complex refractive index of nanoparticles and the host medium, respectively. The optical constants of the materials (Ag, Au and Al) used in this study are shown in Figure S1a–c.

To facilitate the theoretical formulation, the Mie scattering coefficients are advantageously expressed compactly in terms of the Riccati-Bessel functions, which are defined as follows [33]:

(6) $$\psi_{n}\left(\rho \right)=\rho j_{n}\left(\rho \right)$$

(7) $$\xi_{n}\left(\rho \right)=\rho h_{n}^{\left(1\right)}\left(\rho \right)$$

where $j_{n}$ and $h_{n}^{\left(1\right)}$ are the first kind spherical Bessel functions and Hankel functions, respectively.

For the predictive design and analysis of nanoparticle systems embedded with plain spherical nanoparticles, their effective radiative transfer characteristics must be quantified and modeled. The core parameters for this include the extinction coefficient (*μ*_ext_), scattering coefficient (*μ*_sca_), absorption coefficient (*μ*_abs_), and scattering phase function (Φ(*θ*)), which are expressed as follows [34]:

(8) $$\mu_{\mathrm{ext}}=\mu_{p,\mathrm{ext}}+\mu_{m,\mathrm{ext}}=\frac{f_{v}}{{\langle V\rangle }_{r}}C_{\mathrm{ext}}+\frac{4\pi \kappa_{m}}{\lambda }$$

(9) $$\mu_{\mathrm{sca}}=\mu_{p,\mathrm{sca}}=\frac{f_{v}}{{\langle V\rangle }_{r}}C_{sca}$$

(10) $$\mu_{\mathrm{abs}}=\mu_{\mathrm{ext}}-\mu_{\mathrm{sca}}$$

(11) $$\Phi \left(\theta \right)=\Phi_{p}\left(\theta \right)$$

where *f*_v_ is the nanoparticle volume fraction, with 〈*V*〉*_r_* and *κ*_m_ corresponding to the average nanoparticle volume and the imaginary component of the host medium’s refractive index, respectively.

In the theoretical model of the nanofluid filter for the PV/T system investigated in this work, the filter is positioned above the photovoltaic component. It employs spectral splitting to thereby enhance both photoelectric conversion and photothermal collection efficiency. The Monte Carlo model for steady-state light transport in multi-layered tissues (MCML) [35] is employed to solve the radiative transfer equation (RTE), studying radiative energy transfer and consequently understanding the multiple scattering behavior in the theoretical model. The RTE can be expressed as follows [36]:

(12) $$s\cdot \nabla I=\mu_{\mathrm{abs}}I_{b\lambda }-\mu_{\mathrm{ext}}I+\frac{\mu_{\mathrm{sca}}}{4\pi }\int_{4\pi }I\left(\Omega^{\prime }\right)\Phi \left(\Omega^{\prime },\Omega \right)d\Omega^{\prime }$$

where *I* denotes the spectral radiative intensity along direction Ω and path **s**, and *I*_b_*_λ_* is the blackbody spectral intensity. The spectral absorption within the solar cell and the total reflectance are evaluated through the MCML simulation.

The nanofluid filter is positioned at the top of the PV/T system, with its upper interface in contact with the air medium and the bottom adjacent to the glass cover and silicon solar cell. As shown in Figure 1b, the system is irradiated by a vertically incident solar spectrum from above. Through the optical interaction with this nanofluid layer, incident photons are selectively absorbed or transmitted. Based on energy conservation, the spectral transmittance *T*(*λ*), absorptance *A*(*λ*), and reflectance *R*(*λ*) of the system can be determined [36]:

(13) $$R\left(\lambda \right)=\frac{\sum_{2\pi }N_{r}\left(\lambda \right)}{N_{0}\left(\lambda \right)}$$

(14) $$T\left(\lambda \right)=\frac{\sum_{2\pi }N_{t}\left(\lambda \right)}{N_{0}\left(\lambda \right)}$$

(15) $$A\left(\lambda \right)=1-R\left(\lambda \right)-T\left(\lambda \right)$$

where *N*_0_ denotes the incident photon count on the layer, while *N*_r_ and *N*_t_ represent the photons collected by detectors in the hemispherical space above the surface, corresponding to reflected and transmitted photons, respectively. To ensure the convergence and accuracy of the simulation calculations, all Monte Carlo simulations utilized 1 million photon bundles.

Since the standard Fresnel relations in the conventional MCML code are inadequate for interfaces between absorbing media [37], they cannot be directly applied. To address this issue, we have developed an enhanced version of the MCML code that robustly handles reflection and refraction at such interfaces, ensuring an accurate calculation of the nanofluid filter’s optical response.

The reflection laws are first considered for normal incidence. In this case, the reflection coefficient can be formulated using the complex refractive index as follows [38]:

(16) $$r_{∥}=r_{⊥}=\frac{n_{2}-i\kappa_{2}-\left(n_{1}-i\kappa_{1}\right)}{n_{2}-i\kappa_{2}+\left(n_{1}-i\kappa_{1}\right)}$$

For obliquely incident radiation, the reflection coefficients for the polarization components parallel and perpendicular to the plane of incidence are defined by the following equations [38]:

(17) $$r_{∥}=\frac{\cos\theta_{i}/\cos\chi -\left(n_{1}-i\kappa_{1}\right)/\left(n_{2}-i\kappa_{2}\right)}{\cos\theta_{i}/\cos\chi +\left(n_{1}-i\kappa_{1}\right)/\left(n_{2}-i\kappa_{2}\right)}$$

(18) $$r_{⊥}=\frac{\cos\chi /\cos\theta_{i}-\left(n_{1}-i\kappa_{1}\right)/\left(n_{2}-i\kappa_{2}\right)}{\cos\chi /\cos\theta_{i}+\left(n_{1}-i\kappa_{1}\right)/\left(n_{2}-i\kappa_{2}\right)}$$

The reflection of the electric field vector components—parallel (*ρ*_‖_) and perpendicular (*ρ*_⊥_) to the plane of incidence—can be expressed as follows [38]:

(19) $$\rho_{∥}=\tilde{r_{∥}}{\tilde{r_{∥}}}^{*}\;\;\rho_{⊥}=\tilde{r_{⊥}}{\tilde{r_{⊥}}}^{*}$$

where total Fresnel reflection is the average of the two polarized reflections, *ρ* = (*ρ*_‖_ + *ρ*_⊥_)/2. Meanwhile, the absorption index of the absorbing medium affects the calculation of the refractive angle, leading to the complex refractive angle. The complex refractive angle can no longer be interpreted physically as a simple refractive angle for propagation into the material. When materials on both sides of the interface have an absorbing medium, the refraction angle *θ*_2_ can be calculated from the generalized Snell’s law as [36]:

(20) $$p\tan\theta_{2}=n_{1}\sin\theta_{1}$$

where *p* is:

(21) $$p^{2}=\frac{1}{2}\left[\sqrt{{\left(n_{2}^{2}-k_{2}^{2}-n_{1}^{2}\sin^{2}\theta_{1}\right)}^{2}+4n_{2}^{2}k_{2}^{2}}+\left(n_{2}^{2}-k_{2}^{2}-n_{1}^{2}\sin^{2}\theta_{1}\right)\right]$$

The overall performance of the PV/T system is related to the electrical properties of the solar cell and thermal properties of the nanofluid. First, the electrical model of the solar cell was established according to Otanicar et al. [20]. The short circuit current density *J*_sc_ can be written by [39,40]:

(22) $$J_{\mathrm{sc}}=\int_{0.3\;\mu m}^{2.5\;\mu m}G\left(\lambda \right)SR\left(\lambda \right)T\left(\lambda \right)d\lambda$$

where *G*(*λ*) is the AM1.5D solar irradiation spectrum, and *SR*(*λ*) is the spectral response of Si solar cells [41], respectively, as shown in Figure 1c. For Si solar cells, the 700–1100 nm range acts as a core contributing part of the ideal operating wavelength window [39,40]. Therefore, nanofluid-based PV/T systems should transmit sunlight of this part for electrical energy conversion, and absorb energy from other spectral regions for thermal energy conversion.

The dark saturation current density *J*_0_ can be expressed as follows [39,42]:

(23) $$J_{0}=K^{\prime }{T_{c}}^{3/n}\exp\left(\frac{-E_{g}}{mk_{B}T_{c}}\right)$$

where *K*′, *n*, and *m* are empirical parameters, *E*_g_ is the bandgap of the Si solar cells, and *k*_B_ is the Boltzmann constant. *T*_c_ = 298.15 K is the solar cell temperature. The open-circuit voltage, *V*_oc_, can be calculated as follows [40,41]:

(24) $$V_{\mathrm{oc}}=\frac{n_{d}k_{B}T_{c}}{q}\ln\left(\frac{CJ_{\mathrm{sc}}}{J_{0}}+1\right)$$

where *n*_d_ is the diode factor, *q* is the elementary charge, and *C* = 1 is the concentration ratio [41]. In addition, the fill-factor *FF* can be given by [39,41]:

(25) $$FF=\frac{V_{m}}{V_{oc}}\left[1-\frac{\exp\left(\frac{qV_{m}}{k_{B}T_{c}}\right)-1}{\exp\left(\frac{qV_{oc}}{k_{B}T_{c}}\right)-1}\right]$$

where *V*_m_ = *k* × *V*_oc_ is the voltage at the maximum power point, in which *k* is typically between 0.7 and 0.8, and here we set its value to 0.78 [41].

Finally, the electrical conversion efficiency *η*_pv_ of the PV/T system, by solving for the above parameters, can be shown as [39,40]:

(26) $$\eta_{\mathrm{pv}}=\frac{J_{\mathrm{sc}}V_{\mathrm{oc}}FF}{\int_{0.28\;\mu m}^{4.0\;\mu m}G\left(\lambda \right)d\lambda }$$

Afterward, the thermal conversion efficiency *η*_th_ of the PV/T system can be described as [41]:

(27) $$\eta_{\mathrm{th}}=\frac{\eta_{\mathrm{collector}}\int_{0.28\;\mu m}^{4.0\;\mu m}G\left(\lambda \right)\left(1-T\left(\lambda \right)\right)d\lambda }{\int_{0.28\;\mu m}^{4.0\;\mu m}G\left(\lambda \right)d\lambda }$$

where *η*_collector_ = 0.67 is the thermal efficiency of the PV/T collector, which is based on literature values [43].

Finally, in order to evaluate whether different kinds of nanofluids can efficiently convert solar energy into electrical or thermal energy in a PV/T system, the MF is introduced [41]:

(28) $$\mathrm{MF}=\frac{w\cdot \eta_{\mathrm{pv}}+\eta_{\mathrm{th}}}{w\cdot \eta_{\mathrm{pv},\mathrm{unfiltered}}}$$

where the worth factor (*w*) of electrical to thermal energy is assumed to be 3, and *η*_pv, unfiltered_ = 15.89% is the electrical efficiency of the PV/T system without nanofluids [41]. The value *w* = 3 is a commonly adopted factor in PV/T systems, based on the higher economic value of electricity compared to heat. In applications where thermal demand dominates (e.g., district heating), a lower *w* value may be more appropriate. The optimization framework presented herein can readily accommodate different w values by adjusting the MF definition, and the optimal nanofluid parameters would shift accordingly. For consistency with previous benchmark studies [41], we adopted *w* = 3 to enable direct comparison of results.

It should be noted that in this study, the temperature *T*_c_ of the solar cell was fixed at 298.15 K, which is consistent with the standard testing conditions used in reference [41]. In practical operation, spotlight irradiation can cause an increase in battery temperature and affect the photoelectric conversion efficiency through the temperature dependence of the dark saturation current density *J*_0_. An increase in temperature usually lowers the open circuit voltage, leading to a decrease in *η*_pv_. The optical properties (refractive index and absorption coefficient) of nanofluids also vary with temperature, but this effect is usually secondary. The framework proposed in this study can be further extended by using temperature as an additional input parameter to generate simulated data at various battery temperatures, achieving an optimized design considering temperature effects. This will be an important research direction for future work.

### 2.3. Machine Learning Methodology

#### 2.3.1. Decision Tree (DT)

A decision tree is a supervised learning algorithm with a tree-like structure, widely used for classification and regression [44]. It recursively partitions datasets based on feature values to form a tree model. Internal nodes represent feature tests, branches show test outcomes, and leaves represent final decisions or output values. Decision trees are valued for their interpretability, offering a clear decision-making process that is easy to understand [45]. They also adapt well to different data types with minimal preprocessing.

The splitting criterion is central to how decision trees recursively partition data by selecting optimal features. This work utilizes the Gini Impurity as a measure of node purity, selecting the feature that provides the maximum Gini Gain to perform the split. The Gini Impurity of a dataset *D* is defined as [46]:

(29) $$G\mathrm{ini}=1-\sum_{k=1}^{K}P_{k}^{2}$$

where *K* is the number of classes, and *p_k_* is the proportion of samples in dataset *D* belonging to class *k*.

The Gini Gain ΔGini(*D*, *A*), resulting from splitting dataset *D* using feature *A*, is calculated as [46]:

(30) $$\Delta \mathrm{Gini}\left(D,A\right)=\mathrm{Gini}\left(D\right)-\sum_{v=1}^{V}\frac{\left|D^{v}\right|}{\left|D\right|}\cdot \mathrm{Gini}\left(D^{v}\right)$$

where *V* is the number of distinct values for feature *A*, and ∣*D^v^*∣ is the size of the subset of samples in *D* where feature *A* takes the value *v*. The algorithm selects the feature that maximizes the Gini Gain as the splitting feature.

#### 2.3.2. Random Forest (RF)

Random forests are robust ensemble learning tools that aggregate predictions from multiple decision trees, enhancing model accuracy and robustness. This approach mitigates overfitting risks inherent in single decision trees and improves generalization through random feature selection. When dealing with complex datasets, random forests excel by maintaining predictive precision while offering feature importance rankings, which are key to deciphering data structures and drivers.

For regression tasks, the final prediction *f_RF_*(*x*) of the random forest is the average of the predictions from all individual decision trees [47,48]:

(31) $$f_{RF}\left(x\right)=\frac{1}{T}\cdot \sum_{t=1}^{T}f_{t}\left(x\right)$$

where *T* is the total number of decision trees in the forest, and *f_t_*(*x*) denotes the predicted output of the *t*-th tree for the input *x*.

#### 2.3.3. Deep Neural Networks (DNN)

A DNN was employed in this study to establish a high-precision predictive mapping between the geometric parameters of nanofluids [49] and the performance metrics of the PV/T system. The model captures the complex, non-linear relationships by stacking multiple computational layers, wherein the fundamental forward propagation for each layer is governed by the following two equations [50]:

(32) $$Z_{i}^{l}=\sum_{r=1}^{m}\left(w_{ir}^{l}x_{r}^{l-1}+b_{i}^{l}\right)$$

(33) $$a_{i}^{l}=\phi (z_{i}^{l})$$

where *l* denotes the layer index, and *I* represents the index of a specific neuron within that layer, the variable *Z_i_^l^* defines the pre-activation value computed through a linear combination of weighted inputs from the previous layer. Specifically, each weight *w_ir_^l^* multiplies its corresponding activation *a_i_^l^*^−1^ from the *m* neurons in layer *l* − 1, and the sum of these products is adjusted by a bias term *b_i_^l^* that modulates the neuron’s activation threshold. The resulting output activation *a_i_^l^* is then obtained by applying a non-linear activation function *ϕ* to the pre-activation *Z_i_^l^*. Common activation functions include the sigmoid.

### 2.4. Machine Learning Model Evaluation Metrics

The predictive performance of the employed machine learning models (DNN, RF, and DT) for the Ag, Au, and Al nanofluid-based PV/T systems was rigorously quantified using a comprehensive suite of six statistical metrics: the coefficient of determination (R^2^) [26,48], Mean Squared Error (MSE) [48,51], Root Mean Squared Error (RMSE) [48,52], Mean Absolute Error (MAE) [48], Mean Absolute Percentage Error (MAPE), and the Variance Accounted For (VAF) [48]. These metrics collectively provide a multi-faceted assessment of the models’ accuracy and reliability in mapping the complex, non-linear relationships between the nanoparticle geometric parameters (*r*, *f_v_*, *h*) and the target performance outputs (*η*_pv_, *η*_th_, MF).

Specifically, R^2^ gauges the proportion of the variance in the simulated efficiency data that is predictable from the input features [48], serving as a key indicator of how well the learned models capture the underlying physics. MSE and RMSE were prioritized to penalize larger prediction errors [51], which is critical for ensuring the model’s fidelity in capturing the sharp performance shifts near the localized surface plasmon resonance regions, particularly for Ag and Au nanofluids. MAE provides a robust measure of average prediction error magnitude in original efficiency units, enabling direct interpretation of model utility. MAPE complements this by expressing the error as a percentage [53], allowing for a standardized comparison of prediction accuracy across the different efficiency types (photoelectric and thermal) and across the three distinct nanofluids. Finally, VAF was employed as a stringent test of model explanatory power; a VAF value close to 100% indicates that the model’s predictions account for almost all the variability in the simulation data [53], thereby affirming its robustness and suitability for subsequent optimization tasks. The formulae for these evaluation indices are consolidated in Table 1.

### 2.5. Genetic Algorithm (GA)

GA is a powerful evolutionary optimization technique inspired by the process of natural selection. It operates by maintaining a population of candidate solutions, which evolve through iterative applications of selection, crossover, and mutation operators [54,55]. This bio-inspired approach allows GA to efficiently explore complex, high-dimensional search spaces without requiring gradient information, making it particularly adept at handling non-linear, multi-modal optimization problems where traditional gradient-based methods may fail. Its robustness and global search capability have established it as a cornerstone method in computational optimization across diverse engineering fields. In this study, a differential evolution algorithm was employed as the global optimizer, combined with a local refinement step using the L-BFGS-B method. The key parameters are: population size = 30, maximum number of iterations = 200, convergence tolerance = 1 × 10^−6^, mutation factor = 0.8, and recombination probability = 0.5. To avoid local optima, a two-stage strategy was adopted: first, global search with the above settings; second, if the global solution exceeded the best merit function value in the original dataset, a local refinement using L-BFGS-B was performed. The optimization was repeated with 10 different random seeds, and the best result was taken as the global optimum. The population size of 30 was chosen as a balance between search diversity and computational cost.

## 3. Results and Discussion

### 3.1. Model Validation

#### 3.1.1. Radiative Transfer Model Validation

**Figure 3:**
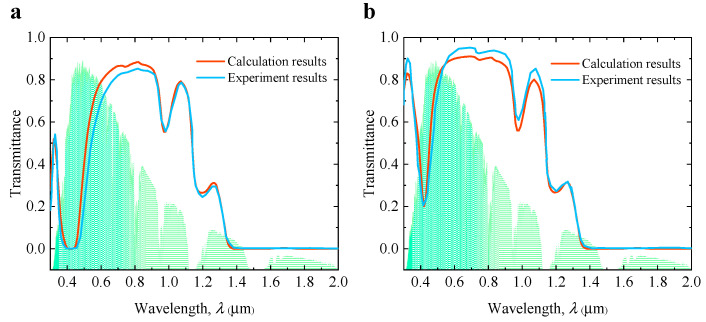
Comparison of calculated and experimental transmittance results of (**a**) Ag-water-2 and (**b**) Ag-water-3 nanofluids with different structural parameters.

To assess the reliability of the proposed radiative transfer model, the transmittances of two Ag-Water nanofluids obtained from the theoretical simulations were compared with the experimental results of Han et al. [41]. The Ag nanoparticles have a diameter of 49.9 nm. The mass fractions of Ag-water-2 and Ag-water-3 nanofluids are 31.8 ppm, as shown in Figure 3a, and 5.3 ppm, as shown in Figure 3b, and the optical path-length is 1 cm. It can be seen from Figure 3 that the transmittances with the simulation agree well with the experimental measurements, which proves the high reliability of the proposed optical model.

In addition to the validation against experimental data presented above, further verification of the core components of the simulation framework is provided in the Supplementary Materials. Specifically, detailed validation results including the comparison of optical properties and the verification of the radiative transfer method are shown in Figure S2 and Table S1, which further confirm the accuracy and reliability of the simulation model.

#### 3.1.2. Model Validation of the PV/T System

The PV/T system model proposed in this study was validated against experimental data from Han et al. [41]. Within the framework of our mathematical model, the electrical and thermal conversion efficiencies of Ag-water nanofluids were calculated using simulated transmittance values and compared with experimental measurements under identical conditions by Han et al. [41]. In their experimental study, Ag-Water-2/3 nanofluid with two mass fractions (31.8 ppm and 5.3 ppm, respectively) was tested. The comparison results are shown in Table 2.

As presented in Table 2, the maximum relative errors between the electrical and thermal efficiencies obtained from our simulations and those from experiments are both under 10%, which falls within an acceptable range. Furthermore, the trends in electrical and thermal efficiency derived from simulations and experiments are consistent. These results demonstrate that the developed mathematical model for the PV/T system exhibits high accuracy, making it suitable for precise evaluation of PV/T system performance.

### 3.2. Performance Analysis of Nanofluid-Based PV/T Systems

#### 3.2.1. The Influence of Geometric Parameters on the SpectralTransmittancee of Nanofluids

**Figure 4:**
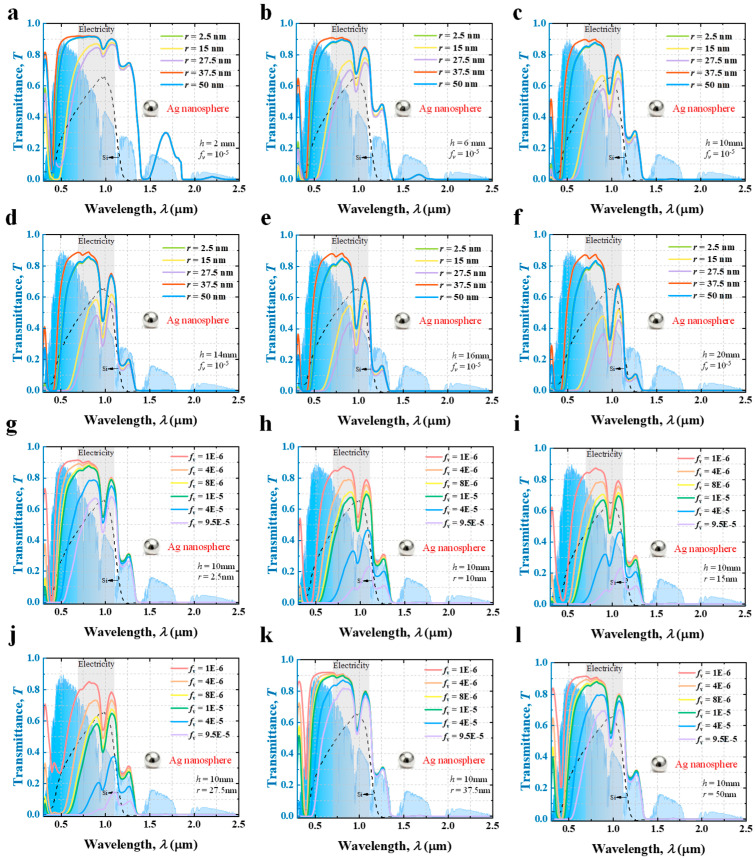
Spectral transmittance of Ag nanofluids as a function of wavelength under various geometric parameters. (**a**–**f**) Transmittance spectra at a fixed low volume fraction (*f_v_* = 10^−5^) with varying nanofluid layer thickness (*h* = 2, 6, 10, 14, 16, 20 mm) and nanoparticle radius (*r* = 2.5, 15, 27.5, 37.5, 50 nm). (**g**–**l**) Transmittance spectra at a fixed layer thickness (*h* = 10 mm) with varying volume fraction (*f_v_* = 1 × 10^−6^, 4 × 10^−6^, 8 × 10^−6^, 1 × 10^−5^, 4 × 10^−5^, 9.5 × 10^−5^) and nanoparticle radius (*r* = 2.5, 10, 15, 27.5, 37.5, 50 nm).

The spectral transmission of nanofluids directly affects the power generation performance of solar panels. Therefore, the influences of geometric parameters on the spectral transmittance of nanofluids are first investigated, as illustrated in Figure 4.

Si solar cells have a significant spectral response in the wavelength range of 400–1100 nm, which can effectively convert incident light in this wavelength range into electrical energy. The 700–1100 nm wavelength range is the core contribution area for achieving photoelectric conversion, so the overall spectral transmittance is required to be relatively high in this wavelength range. As can be seen from Figure 4a, there are significant differences in the spectral transmittance corresponding to different particle sizes. Among them, the spectral transmittance of 37.5 nm particle size is the highest in the 700–1100 nm band, followed by 50 nm and 2.5 nm particle sizes, and the spectral transmittance of 27.5 nm particle size is the lowest. Overall, there is a non-linear relationship between spectral transmittance and particle size. As shown in Figure 4b–f, as the thickness gradually increases (*h* = 6–20 mm), the overall spectral transmittance of Ag nanofluid particles with particle sizes of 37.5 nm, 50 nm, and 2.5 nm in the power generation area changes relatively slightly, while the spectral transmittance of particles with particle sizes of 15 nm and 27.5 nm changes significantly. As the thickness increases, the optical path becomes longer. Due to the characteristic absorption peak of Ag nanoparticles, their spectral transmittance significantly decreases in the wavelength range of 300–400 nm, but has little effect on the subsequent spectral transmittance.

From Figure 4g–l, it can be seen that the spectral transmittance of the nanofluid also has a strong dependence on the particle volume fraction *f_v_*. From the figure, it can be seen that for most different particle sizes, the spectral transmittance in the wavelength range of 700–1100 nm shows a consistent and gradually decreasing trend with increasing concentration. However, the phenomena exhibited by different particle sizes in the figure are not completely consistent. For example, as shown in Figure 4j–l, the spectral transmittance is less affected by increasing concentration at particle sizes of 2.5 nm, 37.5 nm, and 50 nm. In comparison, when the particle size is 37.5 nm, the effect of concentration on it is even smaller. As shown in Figure 4h–j, the spectral transmittance is greatly affected by the increase in concentration at particle sizes of 10 nm, 15 nm, and 27.5 nm. Especially at a concentration of *f_v_* = 9.5 × 10^−5^, the corresponding spectral transmittance is mostly <0.22 in the wavelength range of 700–1100 nm. For the other two nanofluids, Au and Al, although there are differences in details and specific spectral transmittance compared to Ag, their trends and patterns in spectral transmittance with particle size, concentration, and thickness are generally consistent. Please refer to Figures S3 and S4 in the Supplementary Materials for details.

Overall, there is a non-linear and complex relationship between the overall spectral transmittance and the geometric parameters of nanofluids. In the 700–1100 nm wavelength range of concern for PV/T system power generation, as the thickness and concentration increase, it will lead to an increase in nanofluid attenuation, resulting in a general decrease in overall spectral transmittance. The relationship between particle size and spectral transmittance shows a highly nonlinear relationship, which also indicates the need for further in-depth evaluation of the importance of these parameters to the overall system.

#### 3.2.2. The Influence of Geometric Parameters on the Spectral Absorptance of Nanofluids

**Figure 5:**
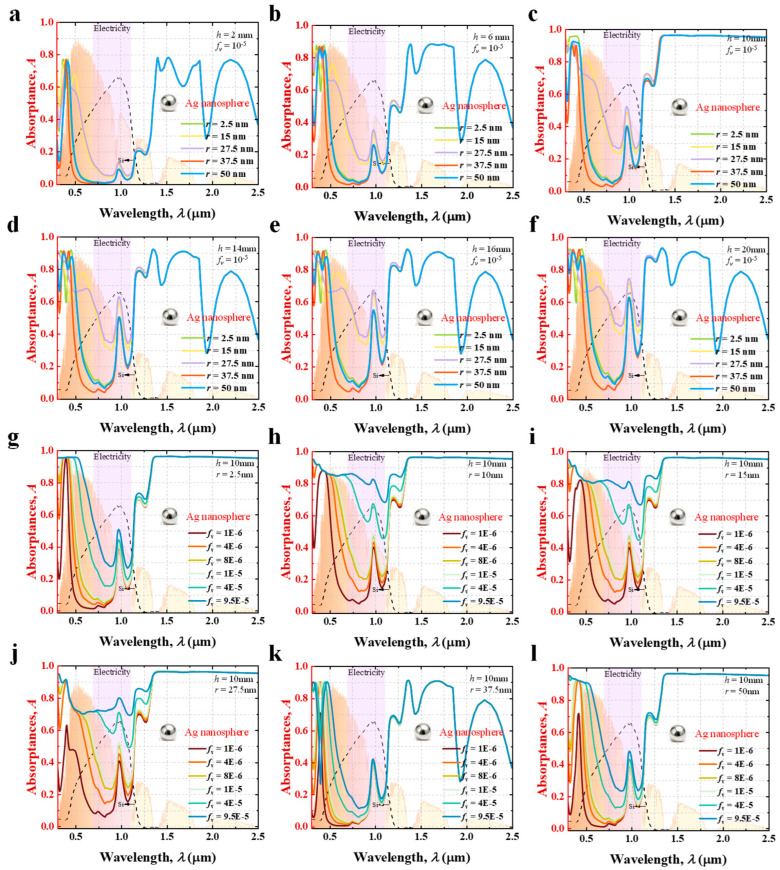
Spectral absorptance of Ag nanofluids as a function of wavelength under various geometric parameters. (**a**–**f**) Absorptance spectra at a fixed low volume fraction (*f_v_* = 10^−5^) with varying nanofluid layer thickness (*h* = 2, 6, 10, 14, 16, 20 mm) and nanoparticle radius (*r* = 2.5, 15, 27.5, 37.5, 50 nm). (**g**–**l**) Absorptance spectra at a fixed layer thickness (*h* = 10 mm) with varying volume fraction (*f_v_* = 1 × 10^−6^, 4 × 10^−6^, 8 × 10^−6^, 1 × 10^−5^, 4 × 10^−5^, 9.5 × 10^−5^) and nanoparticle radius (*r* = 2.5, 10, 15, 27.5, 37.5, 50 nm).

The spectral absorption characteristics of nanofluids directly affect the photothermal efficiency of PVT systems. Figure 5a–f show results at a fixed low volume fraction (*f_v_* = 10^−5^) with varying nanofluid layer thickness *h* and nanoparticle radius *r*, and Figure 5g–l show results at a fixed layer thickness (*h* = 10 mm) with varying particle volume fraction *f_v_* and nanoparticle radius *r*.

To maximize the comprehensive performance of PV/T systems, nanofluids should maintain high transmittance in the response band (700–1100 nm) of Si solar cells to maintain electrical efficiency, while maximizing spectral absorption outside of this band (especially in the short-wave visible and long-wave infrared regions) to improve photothermal efficiency, thus achieving the optimal balance between the two. Therefore, the focus next is on analyzing the effects of spectral absorption, particle size, thickness, and concentration outside the 700–1100 nm wavelength range. As shown in Figure 5a–f, at a fixed *f_v_* = 10^−5^, it can be seen that the spectral absorption outside 1100 nm is less affected by particle size, mainly due to water absorption, while the influence of particle size is mainly reflected in the 300–700 nm wavelength range. From the figure, it can be seen that there are absorption peaks in the spectral absorption curves at different particle sizes, but the corresponding positions have significant differences, which is due to the characteristic resonance absorption of metal nanoparticles. The absorption peak of Ag nanoparticles is between 400 and 500 nm, and as the thickness increases, the effect on the spectral absorption rate is also complex, with the most significant effect occurring between 500 and 1000 nm.

As shown in Figure 5g–l, the spectral absorption of the nanofluid also exhibits a strong correlation with the particle volume fraction *f*_v_. From the figure, it can be seen that under a fixed thickness of 10 mm, the absorption peak positions of the spectral absorption curve of Ag nanofluid at different particle sizes in the 300–500 nm wavelength range are not the same. However, the spectral absorption in the 500–1250 nm wavelength range shows a consistent upward trend with increasing concentration. In comparison, the spectral absorption of particles with particle sizes of 2.5 nm, 37.5 nm, and 50 nm is less affected by increasing concentration. For the main response band of Si solar cell in the wavelength range of 700–1100 nm, it is crucial to choose an appropriate concentration of *f*_v_ and adjust the spectral absorption rate reasonably for both photothermal and photoelectric efficiency, in consideration of power generation. Although the spectral absorptivity of the other two nanofluids, Au and Al, differs from that of Ag in details, their overall trends and patterns with changes in particle size, concentration, and thickness still show a high degree of consistency. Please refer to Figures S5 and S6 in the Supplementary Materials for details.

Overall, similar to spectral transmittance, there exists an extremely complex nonlinear relationship between spectral absorption and the geometric parameters of nanofluids. Therefore, a reasonable selection of geometric parameters is crucial for balancing electrical efficiency and thermal efficiency. Only by achieving collaborative optimization between the two can the comprehensive performance of PV/T systems be maximized.

#### 3.2.3. The Influence of Geometric Parameters on the Performance Outputs (*η*_pv_, *η*_th_, MF)

**Figure 6:**
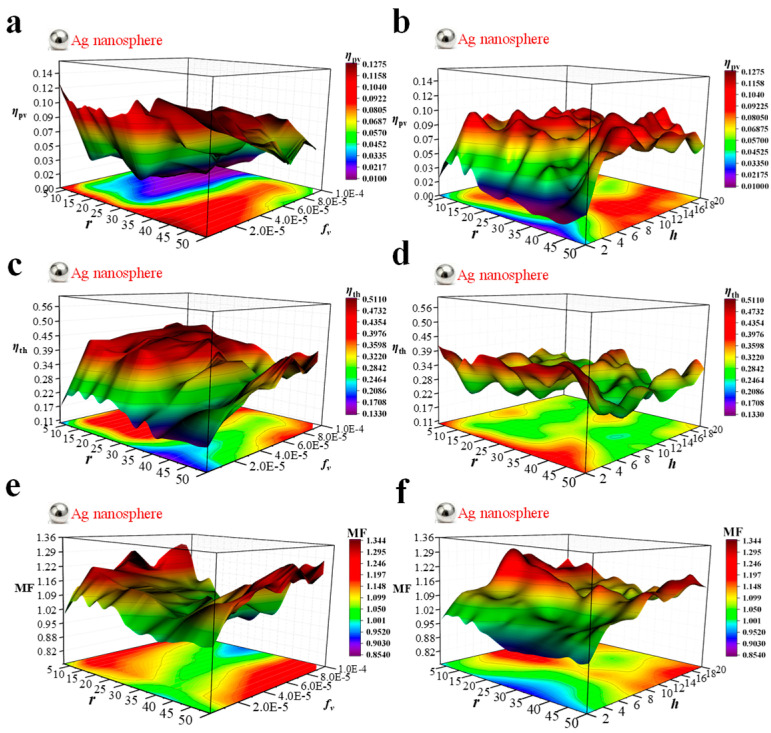
Effects of geometric parameters on the performance of Ag nanofluid PV/T system. (**a**,**b**) photovoltaic efficiency *η*_pv_, (**c**,**d**) thermal efficiency *η*_th_ and (**e**,**f**) merit function MF as a function of particle size *r* versus volume fraction *f*_v_ and particle size *r* versus thickness *h*.

Figure 6 presents the performance outputs (*η*_pv_, *η*_th_, MF) of Ag nanofluid PV/T systems for different geometric parameters (*r*, *h*, *f_v_*). As shown, the variation in performance outputs with geometric parameters is highly nonlinear and extremely complex. As depicted in Figure 6a–d, the effects of nanofluid geometric parameters on the *η*_pv_ and *η*_th_ are generally opposite. The parameter combination conditions corresponding to higher photovoltaic efficiency result in significantly lower solar thermal efficiency. Therefore, when comprehensively analyzing and evaluating the impact of geometric parameters on performance output, special attention should be paid to their influence on MF.

As illustrated in Figure 6e,f, the deep red area (corresponding to the maximum MF value) in the cloud map is distributed on both sides of the cloud map. From Figure 6e, combined with the color mapping bar on the right, it can be seen that when the particle size is in the range of 2.5–25 nm and 45–50 nm, the MF is relatively high. In addition, as can be seen from Figure 6f, when r = 2.5–30 nm, a clear band-shaped region with relatively high MF values appears along the axial direction of the particle size near h ≈ 10 mm. As the volume fraction *f_v_* increases, MF shows an overall upward trend (although there are still fluctuations in the middle layer, and higher values are not necessarily better). These red areas reflect the high-value region of MF. When the geometric parameters fall within this region, the PV/T system can achieve an optimal balance between electric and thermal conversion, while the particle radius can be adjusted within a wide range without significantly affecting the overall performance. For the performance cloud maps of Au and Al nanofluids, please refer to Figures S7 and S8 in the Supplementary Materials for details.

For the entire PV/T system, in order to improve overall system efficiency, the design process should strive to maximize MF and find the corresponding optimal system combination, considering cost-effectiveness. Referring to previous experimental or simulation-based methods to study this problem, if there are relatively few factors considered and operating conditions involved, it may be difficult to find the optimal solution for the system.

### 3.3. Performance Prediction and Optimization Using Machine Learning and GA

**Figure 7:**
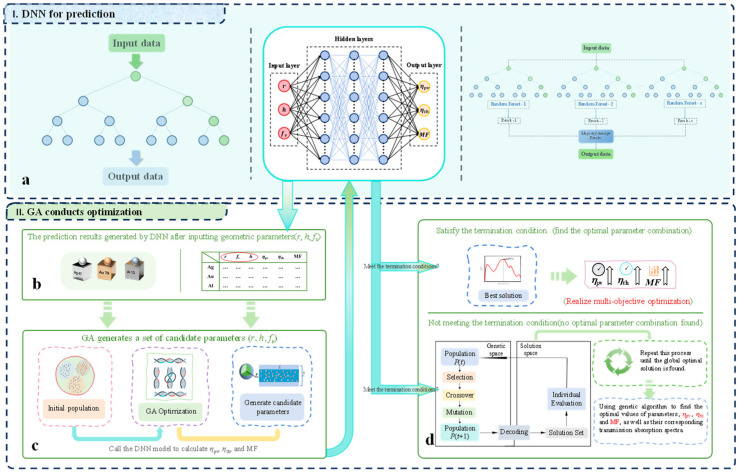
Schematic diagram of the nanofluid PV/T system optimization framework based on a machine-learning-assisted genetic algorithm. (**a**) Model selection and performance prediction. Three machine learning methods, DNN, RF and DT, for performance prediction of nanofluid PV/T system. (**b**) DNN architecture. DNN structure is used to predict performance indicators (*η*_pv_, *η*_th_, MF) from input parameters (*r*, *h*, *f_v_*). (**c**) The GA optimizes the process. The trained DNN is used as the fitness function. (**d**) Operation flow chart of GA. Including selection, crossover and mutation. Finally, the optimal parameter combination of Ag, Au and Al nanofluids was identified.

Figure 7 illustrates the integration framework of machine learning and GA for performance prediction and optimization of nanofluid PV/T systems. As demonstrated in Figure 7a, by comparing the prediction results of DNN, DT and RF models, DNN is finally selected for subsequent prediction. The DNN is trained using datasets to establish the mapping relationship between geometric parameters (*r*, *h*, *f_v_*) and performance indicators (*η*_pv_, *η*_th_, MF), as depicted in Figure 7b. In the optimization stage, the trained DNN is integrated with the GA as a fitness function, as illustrated in Figure 7c. The GA generates a set of candidate parameters, evaluates its MF value through the DNN and approximates the global optimal solution through selection, crossover and mutation iterative evolution, the process of which is outlined in Figure 7d. The framework can efficiently identify the optimal parameter combination of Ag, Au and Al nanofluids.

Furthermore, the proposed prediction and optimization strategy requires only one-time computational and model training costs, offering a simple and fast method for optimizing and predicting nanofluid-based PV/T systems. Detailed information on the machine learning-assisted prediction and genetic algorithm optimization will be presented separately in the subsequent sections.

#### 3.3.1. Machine Learning Methods for Performance Prediction

Accurately predicting and optimizing the performance parameters (*η*_pv_, *η*_th_, MF) of nanofluid-based PV/T systems remains challenging. To address this, this study proposes an integrated strategy combining machine learning methods with genetic algorithms. Initially, high-throughput calculations based on Lorentz-Mie theory and the Monte Carlo method were performed to obtain the spectral transmittance of nanofluids. Based on the mathematical model of the PV/T system, the performance indicators were calculated, and a large-scale dataset was systematically developed, encompassing key geometric parameters. The ranges and intervals of these parameters are provided in Table 3, with the dataset comprising 7400 data points for each material. Then the datasets were split into three groups for training (5920), validation (740), and testing (740) purposes. This dataset provides a solid foundation for subsequent machine learning.

To evaluate the efficacy of various machine learning approaches in predicting the performance metrics of nanofluid-based PV/T systems, three distinct models, DNN, DT, and RF, were trained and tested on an identical dataset. Model performance was rigorously assessed based on its predictive accuracy for photoelectric efficiency (*η*_pv_), thermal efficiency (*η*_th_), and the comprehensive MF.

**Figure 8:**
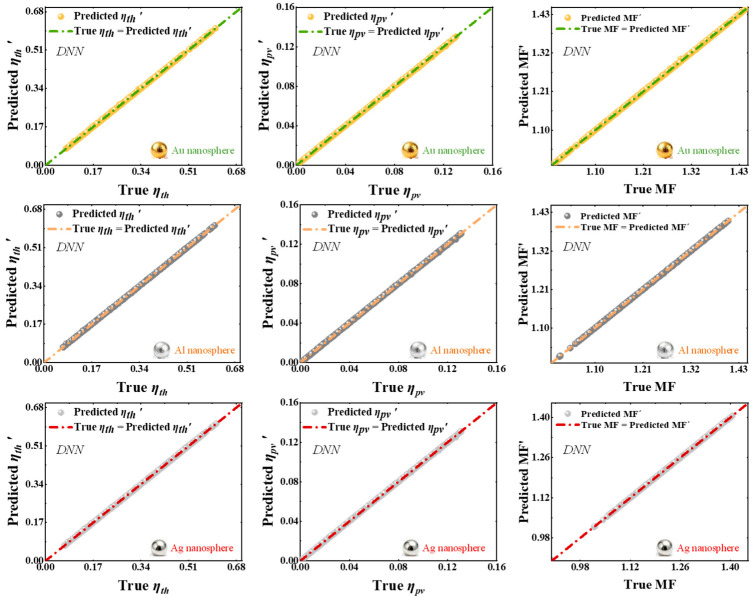
Scatter plots of actual versus predicted values for *η_th_*, *η_pv_* and MF of the three nanofluids under the DNN model.

Figure S9a–i shows the evolution of training and validation loss functions for Ag, Au, and Al nanofluids using DNN, DT, and RF models. All models converged well, with losses decreasing steadily and stabilizing after approximately 1500 iterations. All input and output variables were normalized to zero mean and unit variance using Z-score standardization (Standard Scaler) before training. The dataset was randomly split into training (80%), validation (10%), and test (10%) sets using fixed random seeds. As shown in Figure S9a–i, both training and validation losses decreased steadily and converged after approximately 1500–2000 epochs, with no signs of overfitting. Among them, DNN achieved the best performance, with training and validation losses reduced to 8.76 × 10^−4^ and 4.57 × 10^−5^ after 3000 epochs, respectively. A logarithmic scale is used to better visualize the convergence trends. Figure 8, Figure S10, and Figure S11 present the prediction results for the three-performance metrics of the nanofluids based on the DNN, DT, and RF models, respectively. As shown in the figures, the DNN predictions exhibit excellent agreement with simulated values, confirming their reliability. The DNN achieves the highest prediction accuracy of 99.48%, confirming the reliability of neural network predictions. We acknowledge that explicit L2 regularization and early stopping were not implemented in the current code; this will be considered in future work to further enhance model robustness.

**Figure 9:**
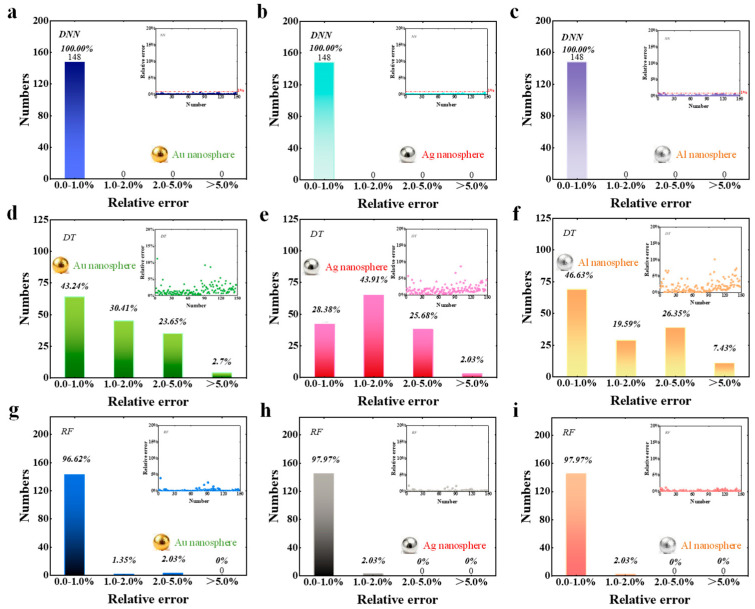
Comparative analysis of machine learning models for nanofluid MF prediction: statistical distributions of relative errors between predicted and actual values, presented in (**a**–**c**) DNN, (**d**–**f**) DT, and (**g**–**i**) RF.

The statistical comparative analysis of the error distribution of the comprehensive evaluation factors in Figure 9 shows significant performance differences between the three machine learning models. As can be seen from Figure 9a–c, the DNN maintained an error accuracy of over 99% in all three types of nanofluid test samples, demonstrating excellent prediction accuracy. In contrast, although the RF model is robust, its accuracy is slightly inferior. As shown in Figure 9g, the sample error accuracy in the Au nanofluid is about 96.62%, while Figure 9h,i show that the error accuracy in the Ag and Al nanofluids is about 97.97%. The DT model exhibits the highest prediction variance, as illustrated in Figure 9d,e, with less than 50% error accuracy in Au and Ag nanofluids, while Figure 9f shows only 46.63% sample error accuracy in Al nanofluids. Overall, the prediction results of these three machine learning models are relatively accurate, and compared to RF and DT, DNN’s prediction results are better.

**Figure 10:**
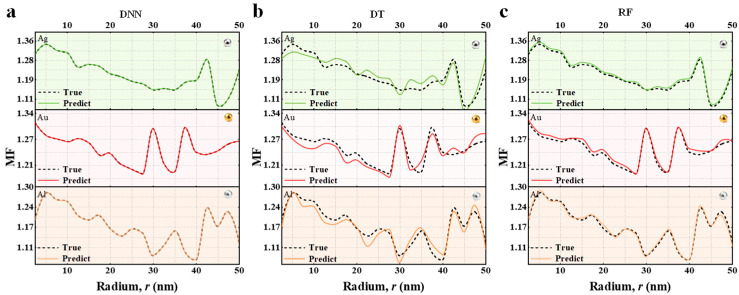
Predicted versus actual MF values for the three nanofluids under the fixed parameter set (*r* = 27.5 nm, *f_v_* = 10^−5^, *h* = 10 mm) are plotted for (**a**) DNN, (**b**) DT, and (**c**) RF models.

Figure 10 shows the comparison curves between the predicted and actual MF values of Ag, Au, and Al nanofluids using DNN, DT, and RF models with fixed geometric parameters (*r* = 27.5 nm, *f_v_* = 10^−5^, *h* = 10 mm). As shown in Figure 10a–c, the curve of the DT model’s predicted values deviates significantly from the actual value curve, with some data showing slight up and down shifts. In contrast, the RF model performs better with slight deviations, while the curves of the DNN model’s predicted values and actual values are approximately perfectly aligned. Although all three models can capture the overall performance trend well, DNN always exhibits better prediction accuracy, once again confirming its optimal position in the comparative methods of this study.

**Figure 11:**
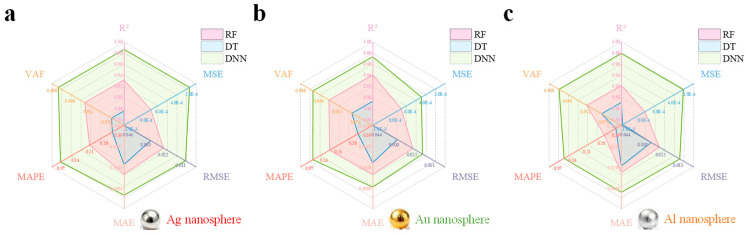
Radar charts of evaluation metrics for the three machine learning models applied to (**a**) Ag, (**b**) Au, and (**c**) Al nanofluids.

Figure 11a–c presents radar charts evaluating the performance of three machine learning models across three types of nanofluids based on multiple dataset metrics. A larger area in the radar chart indicates superior model performance. Through comparative analysis of the models’ results, the DNN model demonstrates the best predictive and generalization capabilities. For instance, in the case of Ag nanofluids on the test set, as shown in Figure 11a, the DNN achieved R^2^ = 0.9864, MSE = 1.37 × 10^−4^, RMSE = 0.0117, MAE = 0.00691, MAPE = 0.09182, and VAF = 0.9865, comprehensively outperforming the other two models, with the RF model being the second best.

The selection of the DNN was driven not merely by its superior final performance metrics, but by its fundamental architectural advantage for this specific regression task. It provides a more accurate and reliable predictive model for the continuous, non-linear performance landscape of nanofluid PV/T systems, effectively circumventing the approximation limitations inherent in tree-based methods. This establishes the DNN model as a more powerful and generalizable tool for the performance prediction and subsequent optimization of such complex energy systems. Consequently, all subsequent critical “prediction–optimization” tasks will be fully developed based on the DNN model.

To accurately capture the complex nonlinear mapping relationship between the geometric parameters of nanofluids (*r*, *h*, *f_v_*) and the performance of PV/T systems (*η*_pv_, *η*_th_ and MF), this study designed and trained a customized feedforward DNN. The core architecture of this network consists of 8 fully connected hidden layers, each containing 2000 neurons, forming a deep model with sufficient parameter capacity and powerful representation ability. The network input layer receives three standardized geometric parameters. Subsequently, the data flows through the eight hidden layers mentioned above, each of which uses ReLU activation functions to introduce nonlinear transformations, which is the key to successfully learning complex physical relationships. Finally, the output layer generates predicted values for three target performance indicators through linear transformation. The detailed training hyperparameters are as follows: the Adam optimizer with a learning rate of 1 × 10^−5^, a batch size of 128, and a total of 3000 epochs. The mean squared error (MSE) was used as the loss function. A MultiStepLR scheduler was applied with milestones at epochs 500, 1000, 1500, and 1800, each reducing the learning rate by a factor of 0.1. The 8-layer architecture was selected based on validation experiments: deeper networks (10–12 layers) exhibited overfitting, while shallower networks (4–6 layers) resulted in lower prediction accuracy. The ReLU activation was chosen to mitigate the vanishing gradient problem. Figure S12 shows the prediction results of the performance evolution of Ag, Au, and Al nanofluids under fixed volume fraction based on the fully trained DNN model and the established “prediction” framework. The model accurately captures key material-specific characteristics.

The computational cost comparison highlights the efficiency of the proposed ML framework. Generating the full training dataset (7400 samples per nanofluid) via conventional physics-based simulations (Lorentz-Mie + Monte Carlo + opto-electro-thermal model) required approximately 70–80 h per nanofluid on an Intel Xeon Platinum 8259CL CPU (2.50 GHz). By contrast, training the DNN (8 hidden layers, 2000 neurons each, 3000 epochs) took only about 2.5–3 h on an NVIDIA GeForce RTX 3090 GPU. Once trained, the DNN predicts *η*_pv_, *η*_th_, and MF for any new set of geometric parameters within <10 milliseconds. This enables rapid GA-driven optimization without the need to rerun expensive numerical simulations for each candidate design, drastically reducing the computational burden of iterative optimization after the one-time training phase.

The verification of this predictive capability underscores the core innovation of our work, which lies in establishing a generalized predictive platform that rapidly and accurately outputs *η*_pv_, *η*_th_, and MF using only basic nanoparticle parameters as input. This approach significantly reduces the reliance on time-consuming and costly experimental trials. DNN offers a high-precision and efficient approach for evaluating solar absorption in complex nanofluids and determining geometric parameters, outperforming traditional time-consuming numerical simulations. Integrating deep learning into this evaluation and design process holds significant potential for advancing photothermal conversion.

#### 3.3.2. Correlation Analysis Among the Key Parameters

**Figure 12:**
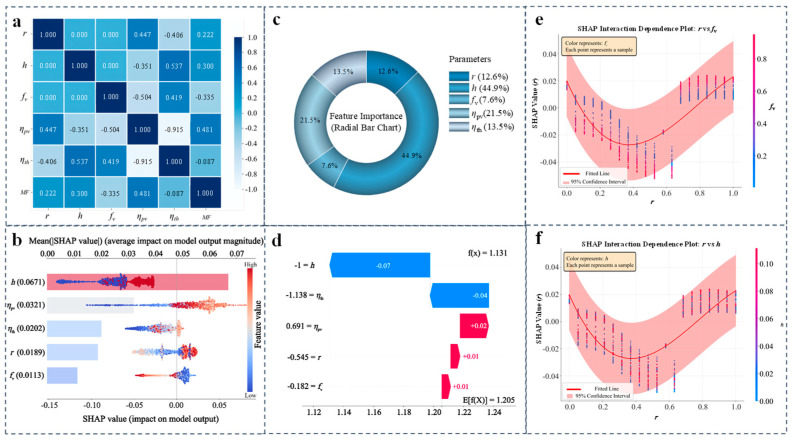
Correlation and SHAP-based interpretability analysis for Ag nanofluid. (**a**) Pearson correlation matrix illustrating linear relationships among input parameters (*r*, *h*, *f_v_*) and output performance metrics (*η*_pv_, *η*_th_, MF). (**b**) Mean absolute SHAP values showing the average impact of each feature on model output magnitude. (**c**) Relative contribution of each feature to the total SHAP importance. (**d**) Waterfall plot demonstrating the decision process for a single sample, with red bars indicating positive contributions and blue bars negative contributions. (**e**) SHAP dependence plot for nanoparticle radius *r* (normalized; original range 2.5–50 nm) with color indicating volume fraction *f_v_*, illustrating the non-monotonic relationship and interaction between *r* and *f_v_*. (**f**) SHAP dependence plot for nanoparticle radius *r* (normalized; original range 2.5–50 nm) with color indicating layer thickness *h*, illustrating the non-monotonic relationship and interaction between *r* and *h*.

To elucidate the contribution of each input feature to model predictions, uncover potential interactions among parameters, and perform a quantitative sensitivity analysis, SHAP analysis was conducted based on the well-trained machine learning model. In this section, taking Ag nanofluid as an example, Figure 12 presents the correlation analysis and SHAP-based interpretability results, including the Pearson correlation matrix, global feature importance, local interpretation, and feature interaction.

Figure 12a shows the correlation matrix between input parameters and performance metrics. These matrices offer a quantitative blueprint that delineates the intricate parameter interactions governing PV/T system performance. A positive value signifies a positive correlation, while a negative value indicates a negative correlation. The further the coefficient deviates from zero, the stronger the association between the two features. The strong negative correlation between *η*_pv_ and *η*_th_ has been confirmed in the matrix diagram (Ag: −0.915). Figure 12b presents the mean absolute SHAP values, illustrating that layer thickness *h* (0.0671) is the most influential feature, followed by *η*_pv_ (0.0321), *η*_th_ (0.0202), *r* (0.0189), and *f*_v_ (0.0113). The relative contribution in Figure 12c shows *h* accounting for 44.9% of total importance, with *η*_pv_ (21.5%) and *η*_th_ (13.5%) also playing significant roles. Figure 12d illustrates the decision process for a randomly selected sample using a waterfall plot. Starting from the base value E[f(x)] = 1.131, layer thickness *h* exerts the strongest negative influence (−0.07), reducing the predicted MF, while *η*_pv_ provides a positive contribution (+0.02). Other features *η*_th_ (−0.04), *r* (+0.01), and *f*_v_ (+0.01)—make minor adjustments, demonstrating that *h* and *η*_pv_ dominate the decision process.

Figure 12e,f illustrates the SHAP dependence plots for nanoparticle radius *r* (normalized; original range 2.5–50 nm). The color in Figure 12e represents volume fraction *f*_v_, while the color in Figure 12f represents layer thickness *h*. The contribution of *r* to MF is non-monotonic, with a slightly positive value in the low *r* region (about 2.5–12 nm) and a gradually negative value in the middle *r* region (about 12–30 nm), reaching its lowest point at about −0.02, while the high *r* region (about 30–50 nm) rebounds and approaches zero. For *f*_v_, high concentrations slightly increase SHAP in the low *r* region, deepen the negative valley in the middle region, and promote recovery in the high *r* region. For *h*, the SHAP of the thick layer slightly decreases in the low *r* region, the negative valley deepens in the middle region, and the recovery is more significantly enhanced in the high *r* region. This indicates that both have a regulatory effect on particle size, and *h* has a stronger influence in the middle and high *r* regions.

#### 3.3.3. Genetic Algorithm for Multi-Parameter Optimization

GA serves as the core optimization engine that drives the design process beyond mere performance prediction. Following the accurate predictions generated by the forward neural network, the GA is employed to efficiently navigate the complex, multi-dimensional parameter space encompassing nanoparticle radius (*r*), volume fraction (*f_v_*), and nanofluid layer thickness (*h*). Its powerful global search capability enables the identification of the optimal combination of these geometric parameters that maximizes the system’s overall MF, a balanced metric of both photoelectric and thermal performance.

Based on the DNN-GA integrated framework shown in Figure 7, the optimal design parameters in Table 4 were obtained through an iterative evolution process. The genetic algorithm first initializes a set of candidate parameter combinations (*r*, *h*, *f_v_*), and quickly evaluates the MF values corresponding to each set of parameters using the trained DNN as the fitness function. Through repeated iterations of selection, crossover, and mutation, the population gradually evolves towards higher MF values, ultimately converging to the global optimal solution. As shown in Table 4, the table is based on the optimal parameter combination obtained through GA optimization.

**Figure 13:**
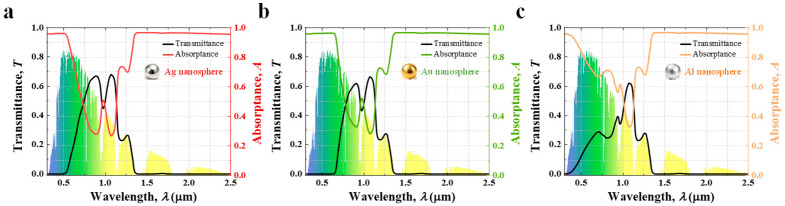
Transmission and absorption spectral characteristics of optimal parameter combinations for (**a**) Ag, (**b**) Au, and (**c**) Al nanofluids after GA optimization.

Figure 13 presents the transmission and absorption spectral characteristics of the optimal parameter combination of Ag, Au, and Al nanofluids after GA optimization. As shown in Figure 13a, the Ag nanofluid with the optimal parameter combination (*r* = 4.02 nm, *h* = 9.91 mm, *f*_v_ = 9.45 × 10^−5^) exhibits the best comprehensive performance, achieving the highest MF of 1.3603 among the three materials. The analysis of its spectral characteristics shows that Ag nanofluid maintains high transmittance within the photovoltaic response range (700–1100 nm), while exhibiting significant absorption characteristic wavelengths in specific bands (300–500 nm, 1300–2500 nm) This ideal spectral modulation capability achieves the optimal balance between thermal efficiency (51.26%) and photoelectric efficiency (4.53%), thereby maximizing the MF value and enabling the PV/T system to achieve the optimal balance between electric and thermal conversion.

Figure 13b shows that the optimal configuration of Au nanofluid (*r* = 2.63 nm, *h* = 10.04 mm, *f*_v_ = 9.67 × 10^−5^) presents unique optimization features. Spectral analysis indicates significant absorption peaks at specific wavelengths, which are characteristic of the plasmonic resonance effect of Au nanoparticles. Although its photoelectric efficiency (4.22%) is slightly higher than that of Ag nanofluid, its excellent thermal efficiency (50.08%) contributes to achieving a final MF of 1.3190. As illustrated in Figure 13c, the optimal parameters of Al nanofluid (*r* = 3.91 nm, *h* = 9.86 mm, *f*_v_ = 9.72 × 10^−5^) exhibit different performance characteristics. The spectral features indicate broadband absorption capability, consistent with its material properties. Despite achieving the highest thermal efficiency (52.93%), its relatively lower photoelectric efficiency (2.57%) results in the lowest MF (1.2978) among the three materials.

Overall, the framework can simultaneously optimize all three geometric parameters while accounting for their complex interdependencies, enabling the discovery of configurations that maximize overall system performance. This represents a significant advancement in nanofluid-based PV/T system design, demonstrating how machine learning-guided optimization can uncover material-specific design rules that fully leverage the unique plasmonic characteristics of each material. In addition, the relationship determined in the Pearson correlation matrix is consistent with the particle characteristics analyzed previously, which further confirms the accuracy of our neural network in capturing the complex correlation between geometric parameters and output performance indicators. Our study emphasizes this machine learning-based prediction and optimization methodology, providing an efficient and reliable pathway for the targeted design and development of high-performance nanofluid filters and establishing a transferable framework for future material screening and system design.

## 4. Conclusions

In this work, an integrated framework combining DNN and GA was established for performance prediction and multi-parameter optimization of nanofluid-based PV/T systems. By leveraging systematic high-throughput computations and data-driven modeling, the research provides an efficient pathway for designing Ag, Au, and Al nanofluid spectral filters. The main conclusions are summarized as follows:

(1) A comprehensive and reliable dataset was constructed through high-throughput theoretical computations integrating Lorentz-Mie theory, Monte Carlo radiative transfer simulations, and a coupled opto-electro-thermal model. The validation results indicated experimental measurements confirmed the model’s high accuracy, with maximum relative errors remaining below 10%, establishing a solid foundation for subsequent machine learning training and analysis.
(2) A rigorous comparative analysis of three machine learning architectures demonstrated the superior predictive capability of the DNN model. It achieved excellent accuracy, achieving a prediction accuracy above 99.48%. In all three performance indicators of nanofluids, they significantly outperform the RF and DT models in capturing the complex, nonlinear structure-performance relationships. SHAP analysis was employed to interpret the model, quantifying feature contributions and identifying nonlinear interactions among geometric parameters, thereby enhancing the framework’s transparency.
(3) A closed-loop prediction–optimization framework was implemented by synergistically integrating the high-fidelity DNN predictor with a GA optimizer. This integrated strategy enables efficient navigation of the complex, multi-dimensional parameter space, drastically reducing the reliance on traditional iterative experimentation and accelerating the discovery of globally optimal design configurations.
(4) Leveraging the combined DNN-GA optimization framework, the global optimum merit function values were identified as 1.3603 for Ag, 1.3190 for Au, and 1.2978 for Al nanofluids, along with their corresponding optimal geometric parameter combinations. This result demonstrates the framework’s powerful capability for the precise identification of global optima and provides specific design parameters to realize the maximum performance potential of different nanofluids.

The major innovation of this work lies in establishing a robust, intelligent design paradigm that seamlessly bridges high-throughput physical modeling with machine learning-driven optimization. This methodology offers substantial potential for accelerating the development of advanced nanofluid-based PV/T systems and provides valuable guidelines for the real-world engineering of high-efficiency solar energy harvesting systems, promising significant reductions in design time and experimental costs. It should be noted that the present study assumes stable dispersion of nanoparticles in the base fluid. In practical applications, nanoparticle agglomeration may occur over time, especially for Ag, Au, and Al nanoparticles. Agglomeration increases the effective particle size and alters the local volume fraction, shifting the spectral transmittance and absorptance, thus deviating from the ideal optimized performance. Previous studies have shown that surface functionalization (e.g., using surfactants such as sodium dodecyl sulfate) or ultrasonication can mitigate agglomeration. Future work will experimentally evaluate the long-term stability of the optimal nanofluid formulations identified in this study and incorporate such effects into the machine learning framework. Furthermore, the proposed framework presents a transferable tool for future material screening and system design in renewable energy applications.

## Figures and Tables

**Table 1:** Evaluation metrics for investigating the performance of three machine learning models.

Evaluation Metrics	Formula
R^2^	$R^{2}=1-\frac{\overset{N}{\underset{i=1}{\sum }}{\left[{{\left(\eta_{pv},\;\eta_{\mathrm{th}},\;\mathrm{MF}\right)}^{\prime }}_{\mathrm{predicted}}-{\left(\eta_{pv},\;\eta_{\mathrm{th}},\;\mathrm{MF}\right)}_{\mathrm{true}}\right]}^{2}}{\overset{N}{\underset{i=1}{\sum }}{\left[{\left(\eta_{pv},\;\eta_{\mathrm{th}},\;\mathrm{MF}\right)}_{\mathrm{true}}-{\left(\bar{\eta_{pv}},\;\bar{\eta_{\mathrm{th}}},\;\bar{\mathrm{MF}}\right)}_{\mathrm{true}}\right]}^{2}}$
MSE	$\mathrm{MSE}=\frac{1}{N}\overset{N}{\underset{i=1}{\sum }}{\left[{{\left(\eta_{pv},\;\eta_{\mathrm{th}},\;\mathrm{MF}\right)}^{\prime }}_{\mathrm{predicted}}-{\left(\eta_{pv},\;\eta_{\mathrm{th}},\;\mathrm{MF}\right)}_{\mathrm{true}}\right]}^{2}$
RMSE	$\mathrm{RMSE}=\sqrt{\frac{1}{N}\overset{N}{\underset{i=1}{\sum }}{\left[{{\left(\eta_{pv},\;\eta_{\mathrm{th}},\;\mathrm{MF}\right)}^{\prime }}_{\mathrm{predicted}}-{\left(\eta_{pv},\;\eta_{\mathrm{th}},\;\mathrm{MF}\right)}_{\mathrm{true}}\right]}^{2}}$
MAE	$\mathrm{MAE}=\frac{1}{N}\overset{N}{\underset{i=1}{\sum }}\left|{{\left(\eta_{pv},\;\eta_{\mathrm{th}},\;\mathrm{MF}\right)}^{\prime }}_{\mathrm{predicted}}-{\left(\eta_{pv},\;\eta_{\mathrm{th}},\;\mathrm{MF}\right)}_{\mathrm{true}}\right|$
MAPE	$\mathrm{MAPE}=\frac{1}{N}\overset{N}{\underset{i=1}{\sum }}\left|\frac{{{\left(\eta_{pv},\;\eta_{\mathrm{th}},\;\mathrm{MF}\right)}^{\prime }}_{\mathrm{predicted}}-{\left(\eta_{pv},\;\eta_{\mathrm{th}},\;\mathrm{MF}\right)}_{\mathrm{true}}}{{\left(\eta_{pv},\;\eta_{\mathrm{th}},\;\mathrm{MF}\right)}_{\mathrm{true}}}\right|$
VAF	$VAF=\left(1-\frac{\mathrm{var}\left({\left(\eta_{pv},\;\eta_{\mathrm{th}},\;\mathrm{MF}\right)}_{\mathrm{true}}-{{\left(\eta_{pv},\;\eta_{\mathrm{th}},\;\mathrm{MF}\right)}^{\prime }}_{\mathrm{predicted}}\right)}{\mathrm{var}{\left(\eta_{pv},\;\eta_{\mathrm{th}},\;\mathrm{MF}\right)}_{\mathrm{true}}}\right)$

**Table 2:** Comparison of simulation results of PV/T system model with experimental results by Han et al. [41].

Type	Electrical Conversion Efficiency (%)		Thermal Conversion Efficiency (%)	
	This Work	Experimental [41]	This Work	Experimental [41]
Ag-Water-2	9.76	9.13	33.63	34.27
Ag-Water-3	12.21	12.29	21.93	22.579

**Table 3:** The dataset and parameter description for three types of nanofluids.

Nanofluid	*r*	*h*	*f_v_*
Au	2.5–50 nm, interval 2.5 nm	2–20 mm, interval 2 mm	5 × 10^−7^–1 × 10^−5^, interval 5 × 10^−7^; 1 × 10^−5^–1 × 10^−4^, interval 5 × 10^−6^
Ag	2.5–50 nm, interval 2.5 nm	2–20 mm, interval 2 mm	5 × 10^−7^–1 × 10^−5^, interval 5 × 10^−7^; 1 × 10^−5^–1 × 10^−4^, interval 5 × 10^−6^
Al	2.5–50 nm, interval 2.5 nm	2–20 mm, interval 2 mm	5 × 10^−7^–1 × 10^−5^, interval 5 × 10^−7^; 1 × 10^−5^–1 × 10^−4^, interval 5 × 10^−6^

**Table 4:** Structure parameter combination and performance metrics after GA optimization.

**Nanofluid**	**Nanofluid Parameters**			**Performance Metrics**		
	*r* (nm)	*h* (mm)	*f_v_*	*η*_th_ (%)	*η*_pv_ (%)	MF
Ag	4.02	9.91	9.45 × 10^−5^	51.26	4.53	1.3603
Au	2.63	10.04	9.67 × 10^−5^	50.08	4.22	1.3190
Al	3.91	9.86	9.72 × 10^−5^	52.93	2.57	1.2978

## Data Availability

The original contributions presented in this study are included in the article/Supplementary Materials. Further inquiries can be directed to the corresponding authors.

## Supplementary Materials

The following supporting information can be downloaded at: https://www.mdpi.com/article/10.3390/nano16110680/s1, Figure S1: Complex refractive indexes of Ag, Au, and Al nanofluids; Table S1: Statistical data for Monte Carlo method validation; Figure S2: Comparison of optical properties between Mie theory and COMSOL 5.6 simulation results, and verification results using Monte Carlo method; Figures S3 and S4: Spectral transmittance of Au and Al nanofluids under varying layer thickness, particle radius, and volume fraction; Figures S5 and S6: Spectral absorptance of Au and Al nanofluids under varying geometric parameters; Figures S7 and S8: Effects of geometric parameters on η_pv_, η_th_, and MF of Au and Al nanofluid PV/T systems; Figure S9: Training and validation loss curves of Ag, Au, and Al nanofluids under DNN, DT, and RF models; Figures S10 and S11: Predicted versus actual values of η_th_, η_pv_, and MF under DT and RF models; Figure S12: DNN-predicted performance metrics of Ag, Au, and Al nanofluids versus particle radius. Refs. [56,57,58,59] are cited in the Supplementary Material file.
