# Supplementary material for: A Novel Prediction-Optimization Machine Learning Framework for Nanofluid-Based Photovoltaic/Thermal Systems
Source: Nanomaterials (Basel). 2026 May 30;16(11):680. doi: 10.3390/nano16110680 (PMC13257623; doi:10.3390/nano16110680)
Supplement: Supplementary file 1 [file nanomaterials-16-00680-s001.zip › nanomaterials-4315830-supplementary.pdf]

Supplementary Materials

A Novel Prediction-Optimization Machine Learning Framework for Nanofluid-based Photovoltaic/Thermal Systems

Chengyuan Li [1], Yankai Huang [2], Zheng Zhang [2], Yan Zhou [1,3], Ruipeng Geng [1], Chengchao Wang [1], Lanxin Ma [1,*]

[1] School of Nuclear Science, Energy and Power Engineering, Shandong University, Jinan, Shandong Province, 250061, China
[2] The Chinese People's Liberation Army Troop 32214, Nanjing, Jiangsu Province, 121000, China
[3] School of Energy and Power Engineering, Nanjing University of Aeronautics and Astronautics, Nanjing 210016, China
* Correspondence: malanxin@sdu.edu.cn

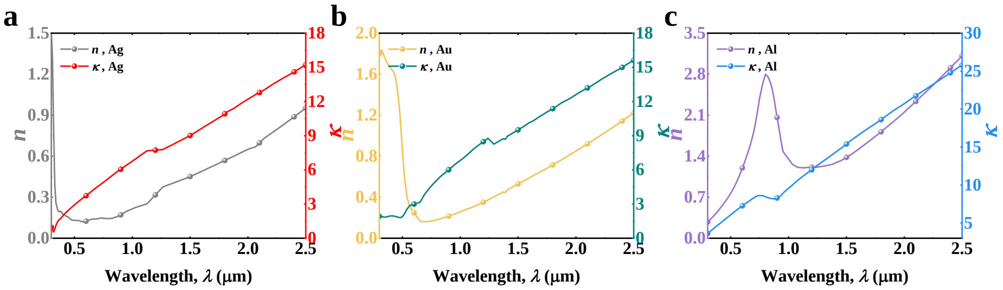

Figure S1. Complex refractive indexes of (a) Ag nanofluid [1], (b) Au nanofluid [2], and (c) Al nanofluid [3].

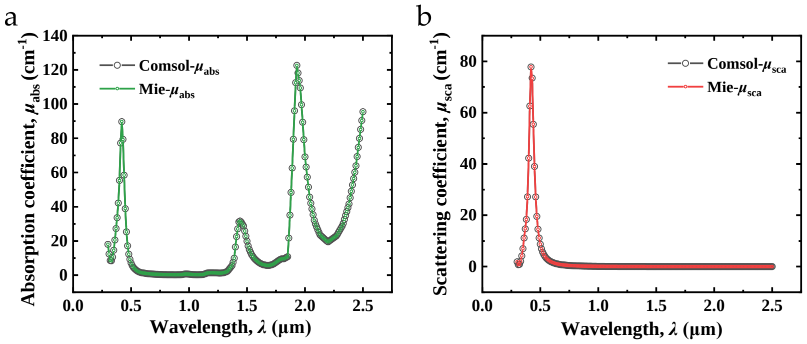

Figure S2. (a) Spectral comparison of absorption coefficients of Ag nanofluids with r = 5 nm calculated by Mie theory and COMSOL simulation. (b) Spectral comparison of scattering coefficients of Ag nanofluids with r = 5 nm calculated by Mie theory and COMSOL simulation.

Table S1. Verification of the total reflectance and transmittance in a slab with a matched boundary.

	R	T
van de Hulst, Ref. [4]	0.09739	0.66096
MC (this work)	0.09735	0.66101
Absolute Error	0.00004	0.00005

To ensure the reliability of the simulation framework, we validated both the optical property calculations and the Monte Carlo (MC) radiative transfer method. The accuracy of the Mie theory-based optical property calculations was verified by comparison with COMSOL Multiphysics simulations. In this work, silver nanofluid was selected as a typical research object for comparative analysis. We calculated the absorption coefficient μ_{abs} and scattering coefficient μ_{sca} of Ag nanofluids with a particle radius of r = 5 nm were computed over the wavelength range of 0.3–2.5 μm. As shown in Figure S2a, b, the Mie theory predictions match the COMSOL results almost perfectly, including the magnitude and spectral location of characteristic absorption and scattering peaks. This confirms the reliability of the optical property data used in the subsequent simulations.

To verify the reliability of the Monte Carlo simulations we compared the computed total reflectance R and transmittance T for a scattering slab against the reference data reported by Van de Hulst [4]. The test case is defined by the following parameters. The relative refractive index n = 1, the absorption coefficient μ_{abs} = 10 cm^{-1}, the scattering coefficient μ_{sca} = 90 cm^{-1}, the asymmetry factor g = 0.75, and the slab thickness h = 0.02 cm. Vertically incident light was considered, and a total of 10^7 photon packets were traced in the simulation. The validation results are presented in Table S1. As shown the computed reflectance R and transmittance T agree very well with the benchmark results from Van de Hulst [4]. These comparisons confirm the accuracy and robustness of the implemented Monte Carlo method for radiative transfer simulations.

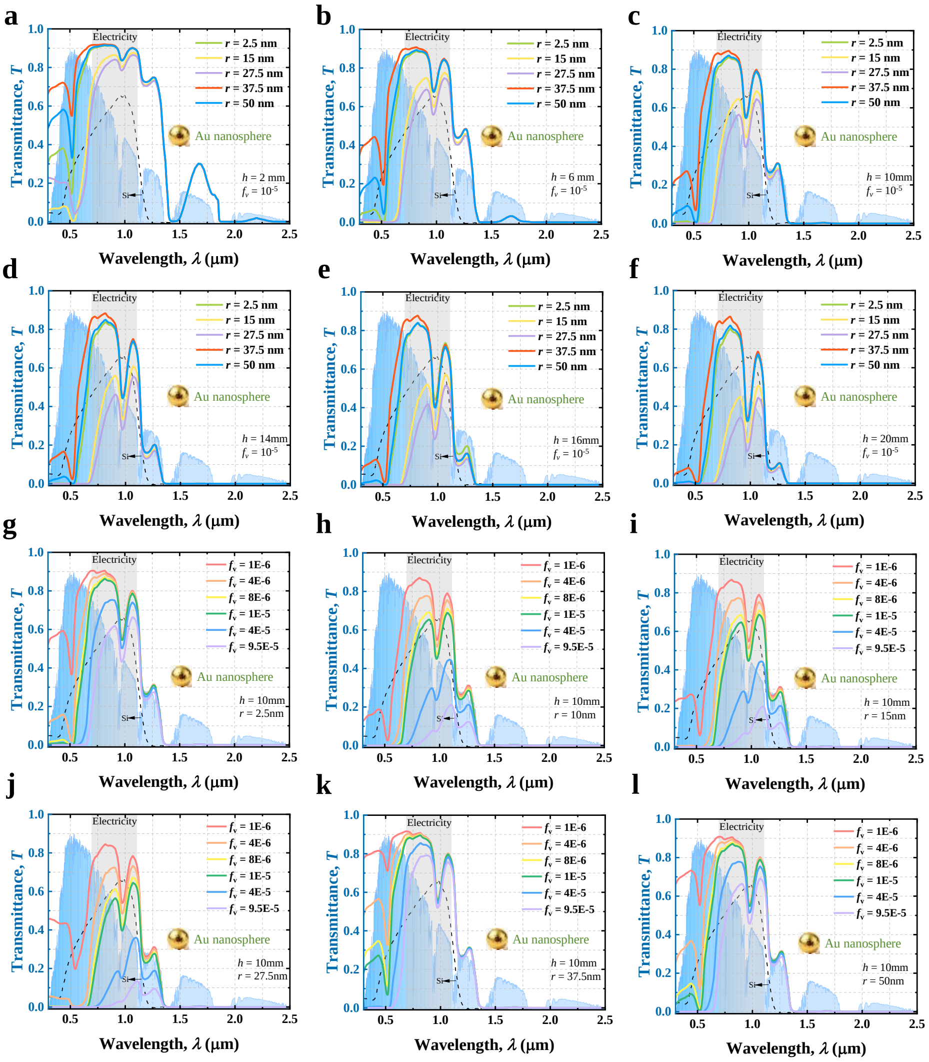

Figure S3. Spectral transmittance of Au nanofluids as a function of wavelength under various geometric parameters. (a-f) Transmittance spectra at a fixed low volume fraction ($f_v = 10^{-5}$) with varying nanofluid layer thickness (h = 2, 6, 10, 14, 16, 20 mm) and nanoparticle radius (r = 2.5, 15, 27.5, 37.5, 50 nm). (g-l) Transmittance spectra at a fixed layer thickness (h = 10 mm) with varying volume fraction ($f_v = 1{\times}10^{-6}, 4{\times}10^{-6}, 8{\times}10^{-6}, 1{\times}10^{-5}, 4{\times}10^{-5}, 9.5{\times}10^{-5}$) and nanoparticle radius (r = 2.5, 10, 15, 27.5, 37.5, 50 nm).

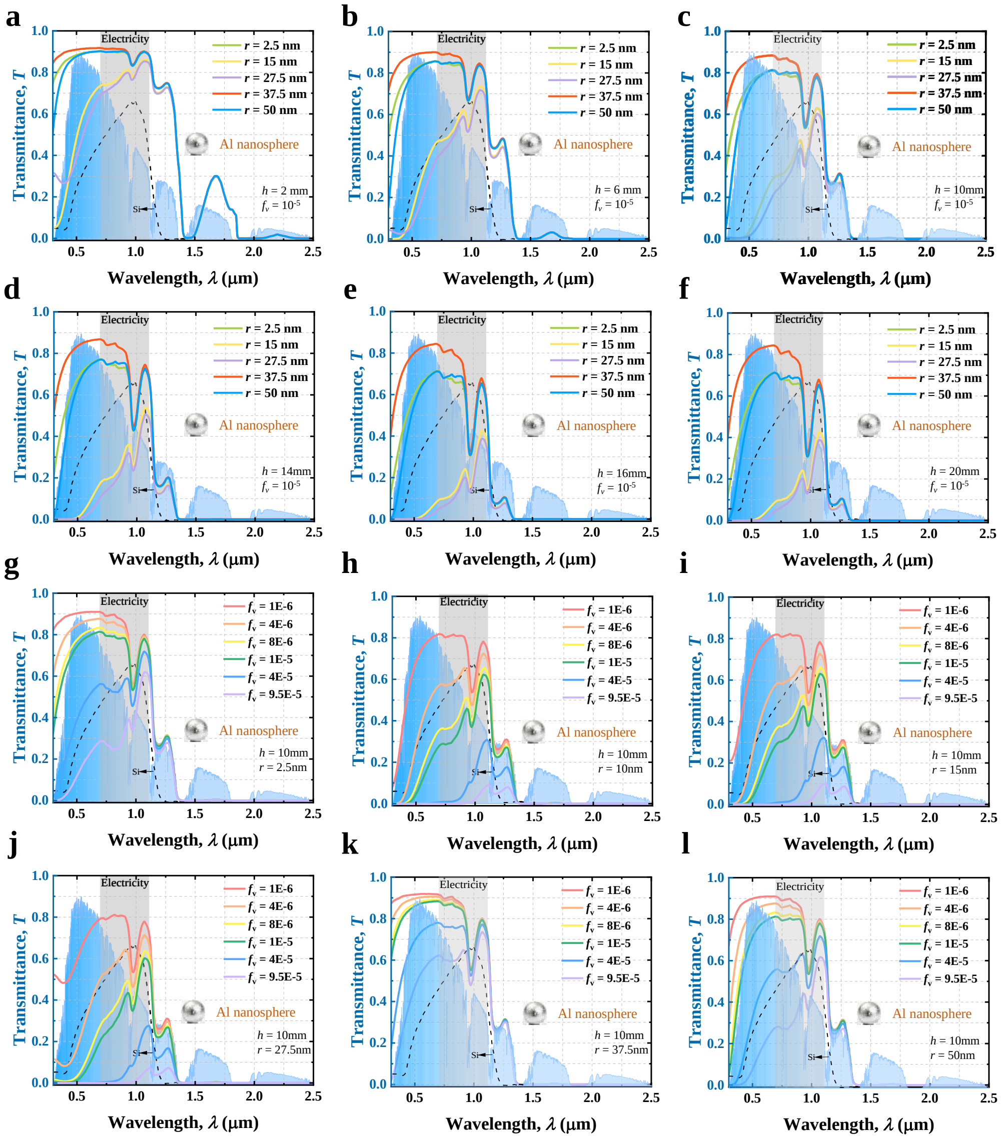

Figure S4. Spectral transmittance of Al nanofluids as a function of wavelength under various geometric parameters. (a-f) Transmittance spectra at a fixed low volume fraction ($f_v = 10^{-5}$) with varying nanofluid layer thickness (h = 2, 6, 10, 14, 16, 20 mm) and nanoparticle radius (r = 2.5, 15, 27.5, 37.5, 50 nm). (g-l) Transmittance spectra at a fixed layer thickness (h = 10 mm) with varying volume fraction ($f_v = 1\times10^{-6}$, 4×10^{-6}, 8×10^{-6}, 1×10^{-5}, 4×10^{-5}, 9.5×10^{-5}) and nanoparticle radius (r = 2.5, 10, 15, 27.5, 37.5, 50 nm).

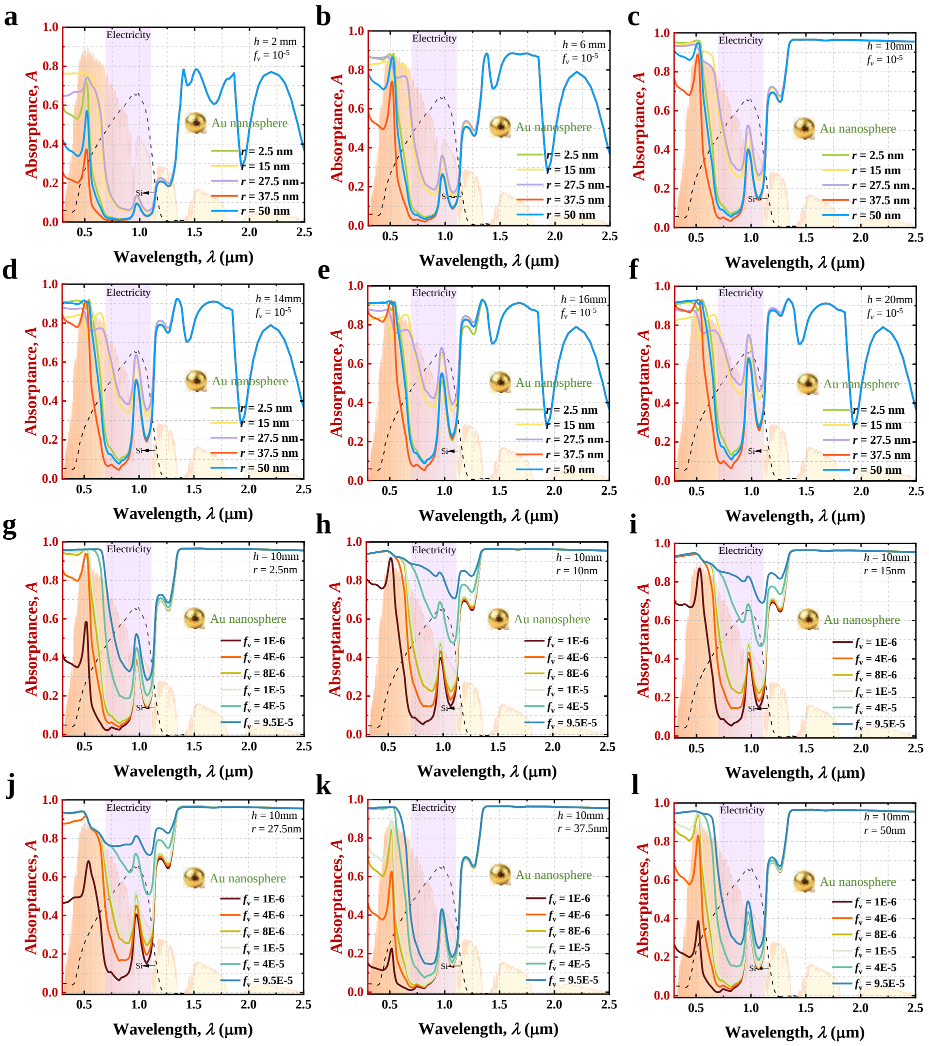

Figure S5. Spectral absorptance of Au nanofluids as a function of wavelength under various geometric parameters. (a-f) Absorptance spectra at a fixed low volume fraction ($f_v = 10^{-5}$) with varying nanofluid layer thickness (h = 2, 6, 10, 14, 16, 20 mm) and nanoparticle radius (r = 2.5, 15, 27.5, 37.5, 50 nm). (g-l) Absorptance spectra at a fixed layer thickness (h = 10 mm) with varying volume fraction ($f_v = 1\times10^{-6}, 4\times10^{-6}, 8\times10^{-6}, 1\times10^{-5}, 4\times10^{-5}, 9.5\times10^{-5}$) and nanoparticle radius (r = 2.5, 10, 15, 27.5, 37.5, 50 nm).

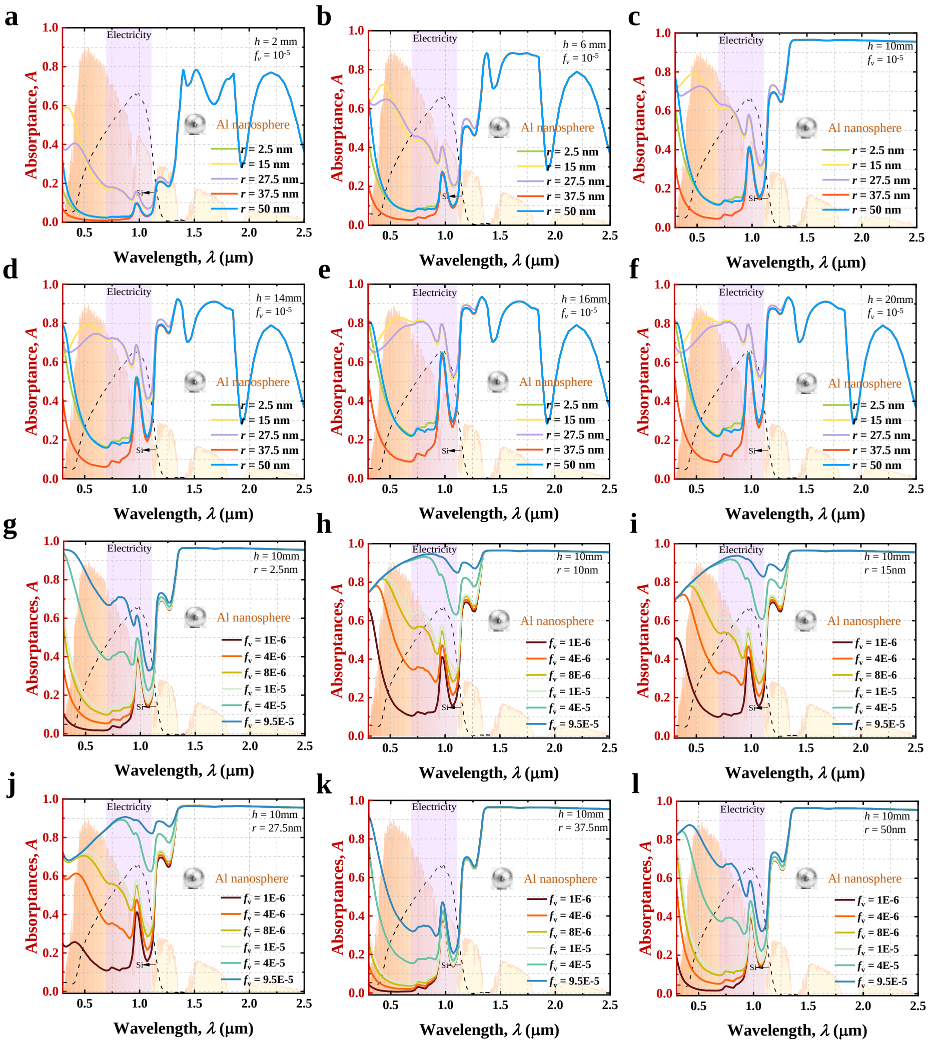

Figure S6. Spectral absorptance of Al nanofluids as a function of wavelength under various geometric parameters. (a-f) Absorptance spectra at a fixed low volume fraction ($f_v = 10^{-5}$) with varying nanofluid layer thickness (h = 2, 6, 10, 14, 16, 20 mm) and nanoparticle radius (r = 2.5, 15, 27.5, 37.5, 50 nm). (g-l) Absorptance spectra at a fixed layer thickness (h = 10 mm) with varying volume fraction ($f_v = 1\times10^{-6}, 4\times10^{-6}, 8\times10^{-6}, 1\times10^{-5}, 4\times10^{-5}, 9.5\times10^{-5}$) and nanoparticle radius (r = 2.5, 10, 15, 27.5, 37.5, 50 nm).

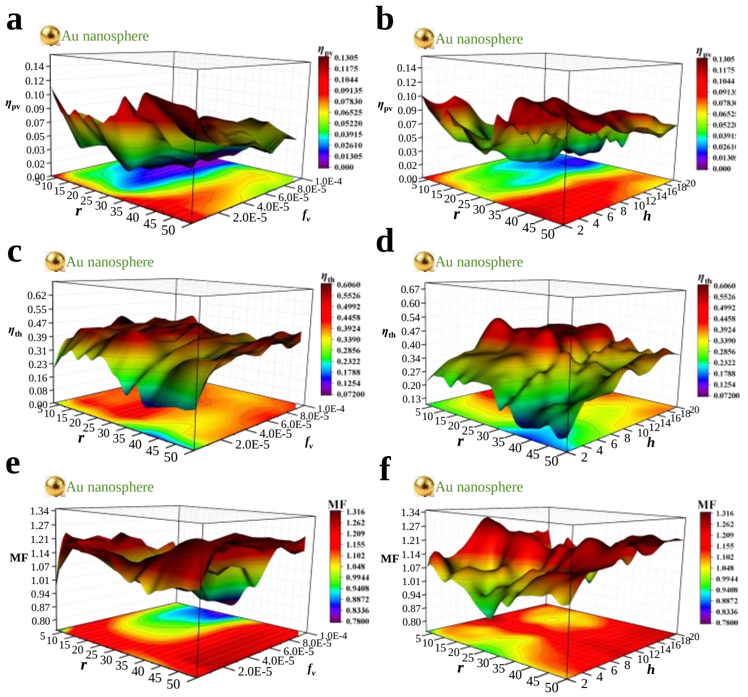

Figure S7. Effect of geometric parameters on the performance of Au nanofluid PV/T system. (a-b) photovoltaic efficiency η_{PV}, (c-d) thermal efficiency η_{th} and (e-f) merit function MF as a function of particle size r versus volume fraction f_v and particle size r versus thickness h.

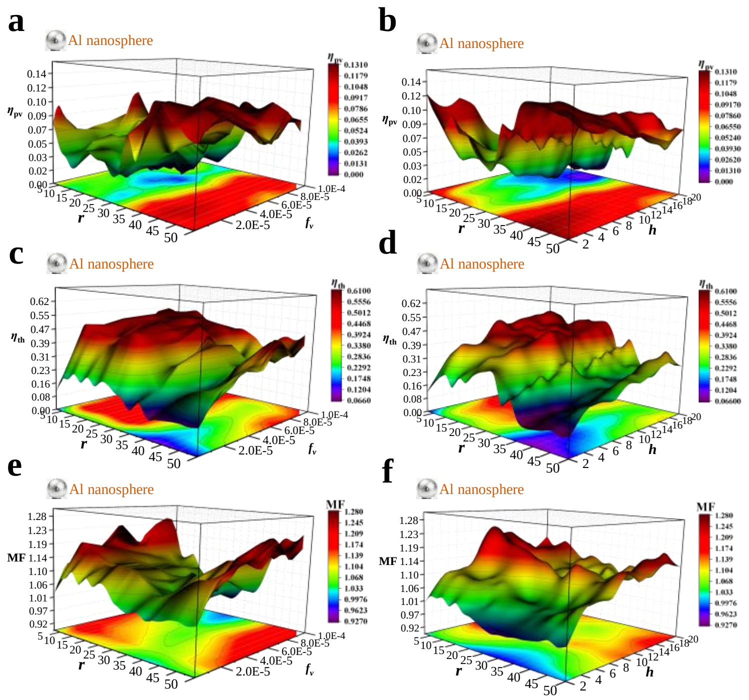

Figure S8. Effect of geometric parameters on the performance of Al nanofluid PV/T system. (a-b) photovoltaic efficiency η_{PV}, (c-d) thermal efficiency η_{th} and (e-f) merit function MF as a function of particle size r versus volume fraction f_v and particle size r versus thickness h.

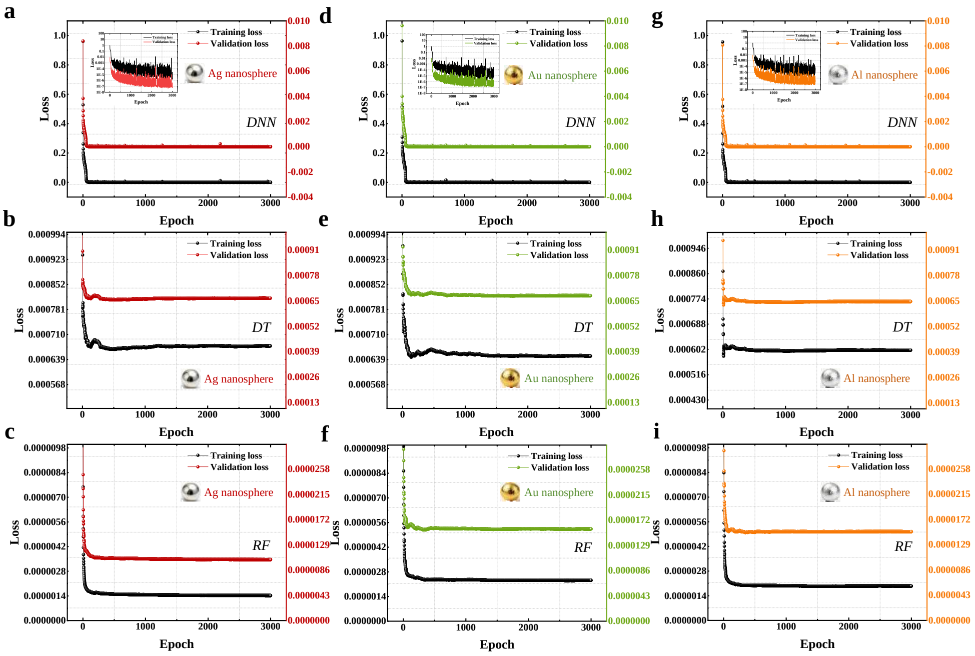

Figure S9. Loss function curves of (a-c) Ag, (d-f) Au, and (g-i) Al nanofluid training and validation sets.

Figure S9 shows the loss evolution of Au, Ag, and Al nanofluids during the training process under DNN, DT, and RF models, respectively. All parameters under the three machine learning models exhibit consistent convergence, with a steady decrease in loss and a stabilization after 1500 iterations.

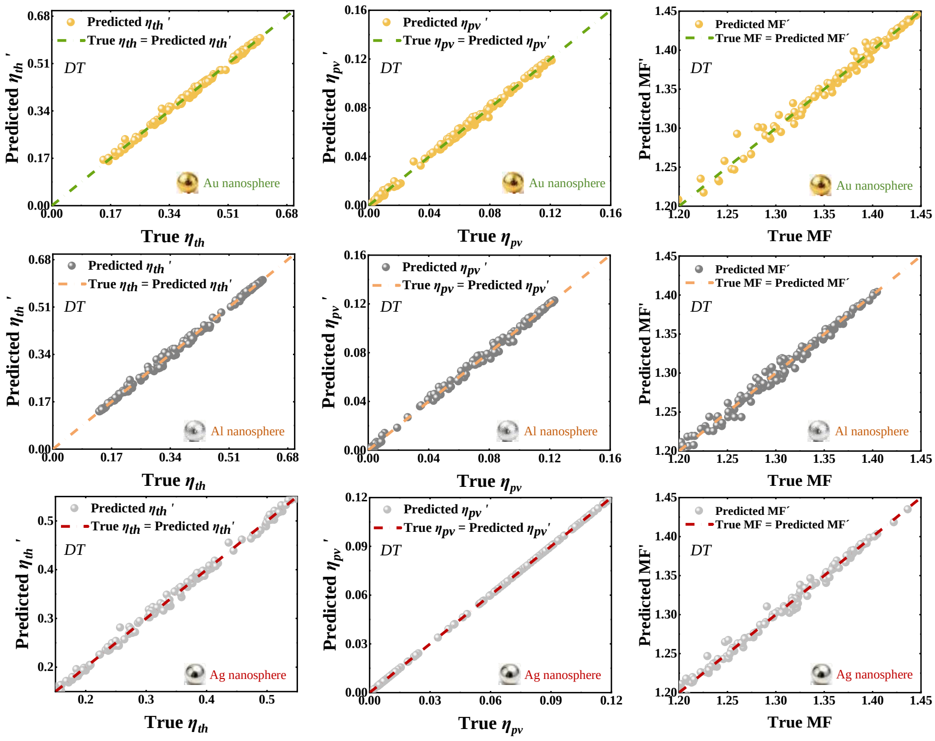

Figure S10. Scatter plots of actual versus predicted values for ηth, ηpv and MF of the three nanofluids under the DT model.

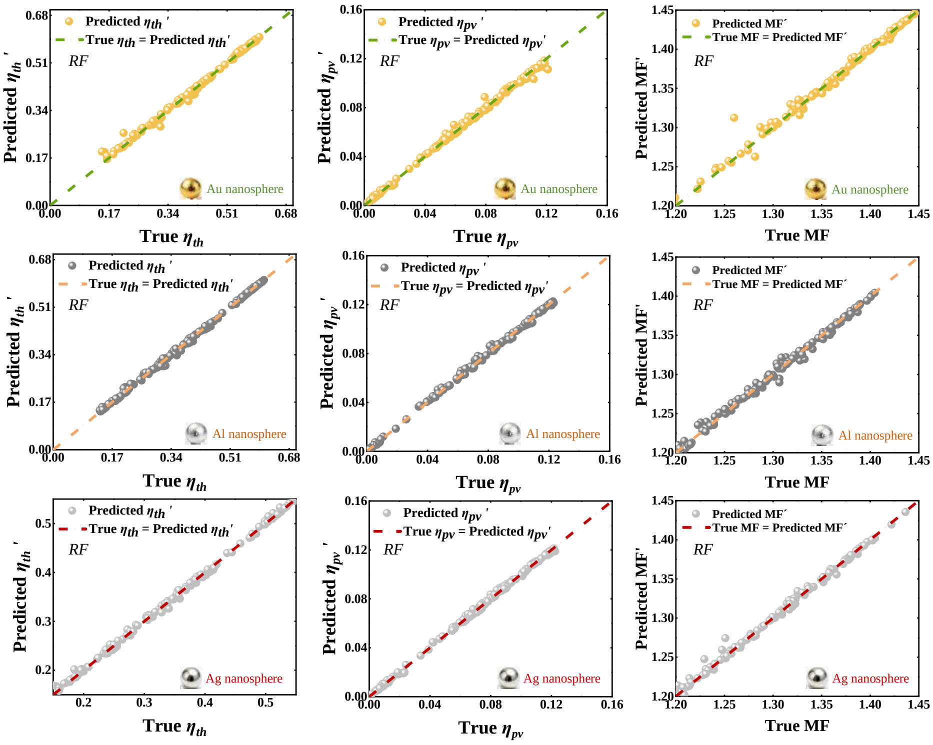

Figure S11. Scatter plots of actual versus predicted values for ηth, ηpv and MF of the three nanofluids under the RF model.

Scatter plots can visually reflect the accuracy of the three machine learning architectures. Figure 8, Figure S10, and Figure S11 present the prediction results for the three-performance metrics of the nanofluids based on the DNN, DT, and RF models, respectively. Through systematic comparison of the three architectures, the DNN demonstrates superior predictive accuracy, achieving a prediction accuracy of 99.48%. This exceptional performance led to the selection of the DNN for predicting the key performance metrics: η_{pv}, η_{th} and MF.

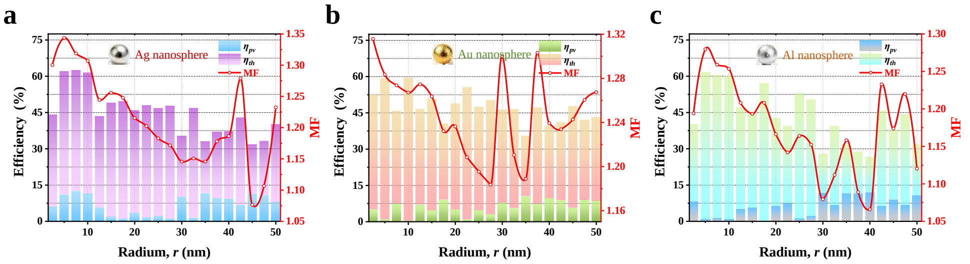

Figure S12. DNN predicted performance metrics for (a) Ag, (b) Au, and (c) Al nanofluids versus nanoparticle radius under $f_v = 10^{-5}$ and $h = 10$ mm.

Figure S12 shows the prediction results of performance evolution of Ag, Au, and Al nanofluids under fixed volume fraction based on the fully trained DNN model and the established "prediction-optimization" framework. The model accurately captures key material specific characteristics. For Ag nanofluids (Figure S12a), it reproduces the distinctive "dual-peak" feature arising from the transition between absorption and scattering dominance. For Au nanofluids (Figure S12b), it captures the performance improvement and resonance red-shift associated with increasing nanoparticle radius. For Al nanofluids (Figure S12c), the model reflects the performance fluctuations and environmental sensitivity inherent to its highly damped plasmonic resonance, realistically mirroring its practical instability. These predictions validate the framework's capability to serve as a generalized platform for rapid and accurate performance forecasting based on fundamental nanoparticle parameters

References

1. Yang, H.U.; D'Archangel, J.; Sundheimer, M.L.; Tucker, E.; Boreman, G.D.; Raschke, M.B. Optical Dielectric Function of Silver. Phys. Rev. B 2015, 91(23), 235137.
2. Johnson, P.B.; Christy, R.W. Optical Constants of the Noble Metals. Phys. Rev. B 1972, 6(12), 4370–4379.
3. Raki, D.A. Algorithm for the Determination of Intrinsic Optical Constants of Metal Films: Application to Aluminum. Appl. Opt. 1995, 34(22), 4755–4767.
4. Van de Hulst, H.C. Multiple Light Scattering: Tables, Formulas, and Applications. Academic Press, New York, 1980.
